# Evolutionary footprint of epistasis

**DOI:** 10.1371/journal.pcbi.1006426

**Published:** 2018-09-17

**Authors:** Gabriele Pedruzzi, Ayuna Barlukova, Igor M. Rouzine

**Affiliations:** Sorbonne Université, Institute de Biologie Paris-Seine, Laboratoire de Biologie Computationelle et Quantitative, Paris, France; University College London, UNITED KINGDOM

## Abstract

Variation of an inherited trait across a population cannot be explained by additive contributions of relevant genes, due to epigenetic effects and biochemical interactions (epistasis). Detecting epistasis in genomic data still represents a significant challenge that requires a better understanding of epistasis from the mechanistic point of view. Using a standard Wright-Fisher model of bi-allelic asexual population, we study how compensatory epistasis affects the process of adaptation. The main result is a universal relationship between four haplotype frequencies of a single site pair in a genome, which depends only on the epistasis strength of the pair defined regarding Darwinian fitness. We demonstrate the existence, at any time point, of a quasi-equilibrium between epistasis and disorder (entropy) caused by random genetic drift and mutation. We verify the accuracy of these analytic results by Monte-Carlo simulation over a broad range of parameters, including the topology of the interacting network. Thus, epistasis assists the evolutionary transit through evolutionary hurdles leaving marks at the level of haplotype disequilibrium. The method allows determining selection coefficient for each site and the epistasis strength of each pair from a sequence set. The resulting ability to detect clusters of deleterious mutation close to full compensation is essential for biomedical applications. These findings help to understand the role of epistasis in multiple compensatory mutations in viral resistance to antivirals and immune response.

## Author summary

Epistasis is a widespread and ubiquitous genetic property of biological networks that shape evolutionary trajectories. Detecting epistasis in genomic data still represents a great challenge that could be met with a better understanding of epistasis from a mechanistic, evolutionary point of view. Here, using a standard model of population genetics applicable to viruses and microorganisms, we derive a universal relationship between four haplotype frequencies of a single pair in a genome, which depends only on the epistatic strength for the pair, defined regarding Darwinian fitness, but not on other system's parameters. Although we do not yet propose a tool for statistical inference of epistasis, we derive an analytic estimator of its strength from a single time DNA/RNA sequence database. The method is based on the balance existing between fitness and disorder caused by mutation and random genetic drift. Our technique can be used to understand the mechanism behind the rapid accumulation of mutation clusters that can cross the threshold of genetic stability and cause rapid adaptation of a virus to a new host, a new antiviral drug, or a new wave of immune response.

## Introduction

Abundant evidence demonstrates that epistasis plays a vital role in the genetic evolution of populations and the heritability of complex traits. In a biological system, each protein, DNA or RNA could serve multiple functions and interact with several partners. The term epistasis [1, 2], which refers to these biochemical interactions, is a widespread property of biological networks [2, 3, 4] and a subject of intense studies. The inclusion of epistatic contributions has been shown to improve phenotype predictions in model organisms including chicken [5, 6], yeast [7–9], and various plants [10–12]. The literature on human diseases is abundant with reports of epistatic interaction for thousands of pairs of single nucleotide polymorphisms (SNPs) in humans [13, 14] pointing at a major role for epistasis in the genetics of human diseases [15–23].

Although the number of proposed epistatic interactions is extensive, few of them are agreed upon [4, 24]. It appears that epistasis hides among millions of possible SNPs pairs at the genome level. Quite a few existing search techniques employ statistics and information theory to infer SNP interactions [25–30]. They range from regression analysis [31, 32] to Bayesian techniques [33–35] to methods based on linkage disequilibrium (LD) and haplotype statistics [36, 37]. All of them estimate epistasis based on inter-locus pairwise association frequencies. Despite the variety of measures, detecting epistasis remains a statistical challenge, and few if any reports of statistically-defined epistasis are reproducible and experimentally validated [3, 4].

Apart from the statistical problem of data noise, these efforts are impeded by the lack of a measure of interaction that would define epistasis in a specific biological context and separately from other system parameters. All the existing methods use statistical markers [3, 4], which are designed to infer interacting pairs but not the strength of interaction separate from other parameters and state variables. A crucial biological scenario that requires a better theoretical understanding of the nature of epistatic effects is a viral population evolving through sudden changes in selection pressure. Evolutionary bottlenecks occur during the viral transmission to a new host, the spread to a different organ or coping with a new therapeutic agent. Viral populations are characterized by a high genetic diversity due to large mutation rates, short generation spans, and relatively large population sizes. Viral populations can re-adapt very quickly to sudden changes in environment. Often, an adapting virus passes through intermediate genetic variants with reduced fitness, termed "fitness valleys" [38, 39, 40, 41]. Compensatory epistatic mutations can rescue replicative fitness while preserving the resistant phenotype [38, 39, 42–44].

A better understanding of how epistasis affects evolutionary trajectories would help to predict compensatory epistatic interaction expediting the development of drug resistance [40]. Rather than acting directly, epistatic compensatory mutations may represent allosteric sites which act indirectly [45], and they cannot be inferred based on structural studies. Experimental proof of compensatory epistatic interaction rescuing viral fitness has been found [38–40, 42–46]. A recent example is a pair of mutations E138K and M184I conferring cross-resistance against four FDA-approved drugs in phase 3 clinical trials linked to HIV treatment failure [46]. The pair 150L and A71V is also associated with drug resistance [47, 48]. The epigenetic pairs close to full-compensation pre-existing in the viral genomic pool are sorted out and eventually develop drug resistance [48]. To accurately predict these critical epistatic pairs, it is essential to understand how epistasis affect evolutionary trajectories and to identify its fingerprint at the genetic level.

In the present work, we define a measure of epistasis in Darwinian terms and predict its genetic signature from mechanistic analysis of epistatic effects on genetic diversity. Specifically, we model the stochastic evolution of a haploid population in the absence of recombination within a broad time interval, in the presence of selection, random drift, and mutation. We focus on the case of positive epistasis in a diverse population evolving after a sudden change of environment. For the general case of a pair of loci linked to a long genome, we obtain a relationship between haplotype frequencies that depends only on the strength of epistatic interaction for the given pair. Through theoretical derivation and simulation, we prove that our measure of epistasis is relatively independent of the underlying topology, the state of the population, and model parameters. We also discuss possible caveats and the potential applicability of our method as a tool to identify the genetic signature of epistatic interaction involved in drug resistance.

### Model

Here, we consider a haploid population of *N* binary sequences of {*K*_*i*_}, where each genome site (nucleotide position) numbered by *i* = 1, 2, …, *L* is either *K*_*i*_ = 0 or *K*_*i*_ = 1. We assume that the genome is long, *L* >> 1. Evolution of the population in discrete time measured in generations is simulated using a standard Wright-Fisher model, which includes the factors of random mutation with genomic rate μ*L*, natural selection, and random genetic drift [51–60]. Recombination is assumed to be absent. Once per generation, all individuals die and are replaced with their progeny, whose number is random and obeys a multinomial distribution. The total population stays constant with the use of the broken-stick algorithm. We included natural selection as the average progeny number (Darwinian fitness) of sequence {*K*_*i*_} is set to *e*^*W*^ where

$$W=\sum_{i=1}^{L}s_{i}K_{i}+\sum_{i<j}^{L}s_{ij}K_{i}K_{j}$$ (1)

$$s_{ij}=E_{ij}(\left|s_{i}|+|s_{j}\right|)T_{ij}$$ (2)

The first term in Eq 1 stands for the additive contribution of single mutations to fitness with selection coefficients *s*_i_. The second term in Eq 1 describes pairwise interactions of sites with magnitudes *s*_*ij*_ given by Eq 2. Coefficient *E*_*ij*_ represents the relative strength of epistatic interaction between sites *i* and *j*, while the binary elements of matrix **T** = {*T*_*ij*_} indicate interacting pairs with *T*_*ij*_ = 1 and the non-epistatic pairs by 0. Note that if we consider an isolated pair of two deleterious mutations, by definition, *E*_*ij*_ = 1 corresponds to *W* = 0 in Eq 1, i.e., full mutual compensation of deleterious mutants at sites *i* and *j*.

We note that there are different definitions of the sign of epistasis in the literature. In the present work, we set the sign of epistasis *E*_*ij*_ to be the same as the sign of the interaction term *s*_*ij*_, regardless of the signs of selection coefficients. If interaction increases fitness, we have *E* > 0 (positive epistasis), and if it decreases fitness, we have *E* < 0 (negative epistasis). In the case when *s*_*i*_ and *s*_*j*_ have opposite signs, according to Eq 2, the resulting interaction term *s*_*ij*_ of the epistatic pair is also positive.

## Results

### Positive epistasis affects the accumulation of deleterious mutations

To understand the general effect of epistasis on the speed of evolution, we simulated a population of genomes, initially 100% wild-type: all *K*_*i*_ = 0. In general, the distribution of selection coefficient over sites is somewhat complex. We considered four most uncomplicated cases, where all selection coefficients *s*_*i*_ in Eq 1 are either negative or positive and their absolute values are fixed, *s*_i_ = *s*_0_, and so is epistatic strength *E*_*ij*_ = *E*. Interacting pairs defined by matrix {*T*_*ij*_} were chosen randomly, on average, with one interaction per site (1/*L*)∑_*ij*_*T*_*ij*_ = 1. We considered two measures of evolution speed, the adaptation rate, *A* = (1/*L*)*dW*/*dt*, and the substitution rate, *V* = *df*/*dt*, where *f* is the mutation frequency per site, and averaged them over a short time interval before equilibrium. The value of *V* represents the rate at which mutations are added to the population, and *A* is the rate of fitness change ("fitness flux").

**Fig 1:**
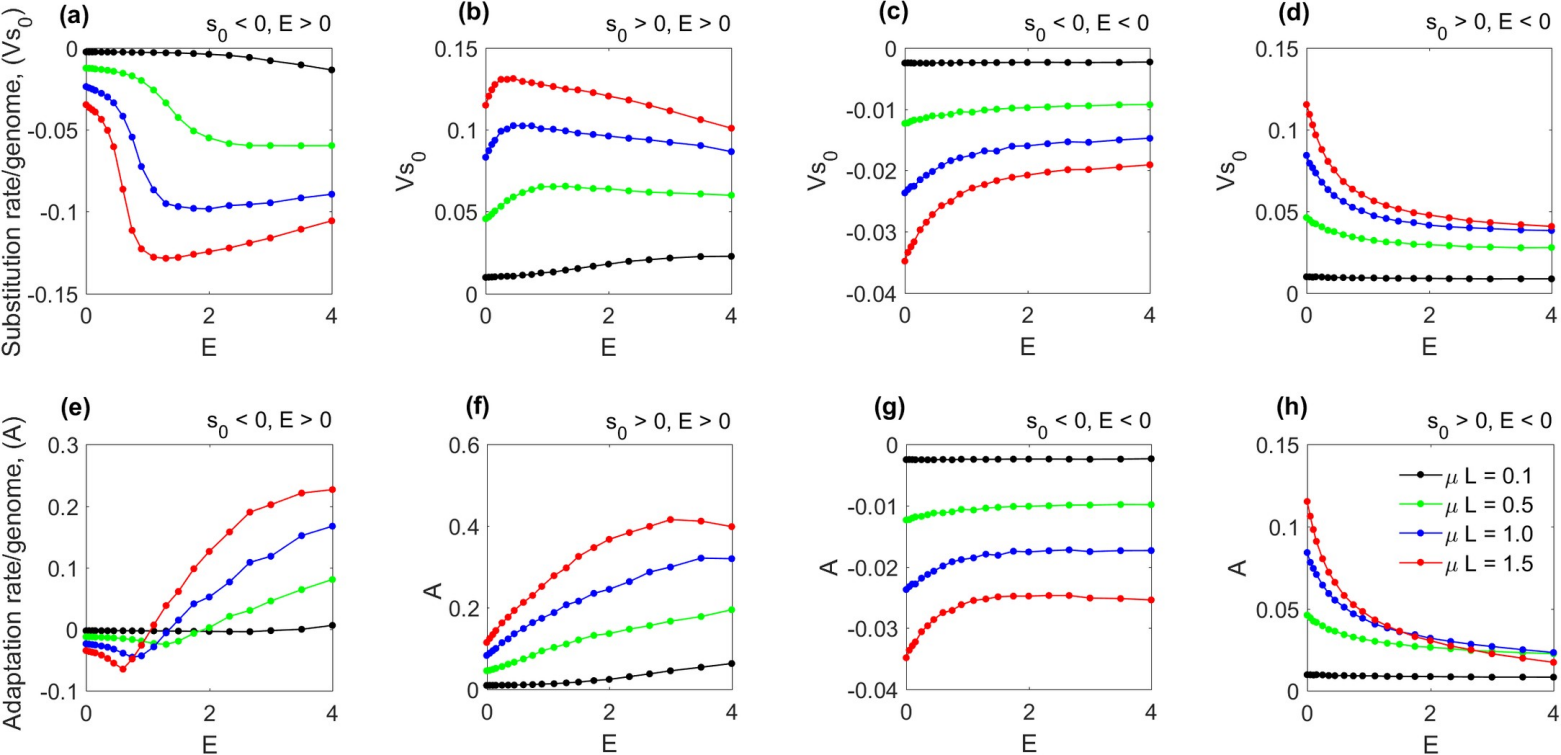
Positive epistasis enhances adaptation. Wright-Fisher population of 500 genomes has been simulated for 20 generations, starting from uniformly wild-type (best-fit at *E* = 0) population. The adaptation rate *A* = *dW*/*dt* (bottom row) and substitution rate *Vs*_0_ = *s*_0_ *df*/*dt* (upper row) were averaged over 300 runs and are plotted as a function of the epistatic strength, *E*. The selection coefficient *s*_0_ and *E* are the same for all sites. Parameters in (a-h): |*s*_0_| = 0.2, total site number *L* = 300, mutation rate per genome μ*L* is shown (colors). The binary connectivity matrix *T*_ij_ is random with ~1 interaction per site. Each column corresponds to a different sign of *s*_0_ and *E* (shown). (a, d, e, h) In the two cases of reciprocal epistasis, the evolution rates demonstrate strong non-linear dependence on the epistatic strength.

Positive epistasis (*E* > 0) significantly enhances accumulation of deleterious mutations (Fig 1A and 1E). In this case, the adaptation rate changes sign when passing through the point of full compensation, *E* = 1. In this interval, coupled pairs of mutations become beneficial for genome fitness, even though single mutations are deleterious. An example of this case is observed in Fig 1B and 1F. Positive epistasis increases the adaptation rate of beneficial mutations as well, but its effects on substitution rate V are modest.

Counterintuitively, positive epistasis may decrease substitution rate for positive alleles (Fig 1B). Indeed, the fitness of a genome depends not only on the number of alleles but also on the proportion of paired interacting alleles. A pair of alleles has a larger fitness gain than two unpaired alleles have together. Thus, genomes with a smaller number of paired positive alleles outcompete the ones with a larger number of unpaired alleles (Fig 1A and 1E).

Negative epistasis, for any sign of *s*_0_ (Fig 1C, 1G, 1D and 1H), has a relatively weak effect: if the mutation rate is very large, both substitution and adaptation rates are decreased by absolute value. Below we focus on the most interesting case of partial compensation (Fig 1A and 1E; *E* > 0, *s*_0_ < 0). Instead of short-term adaptation, we will consider evolution on long timescales.

### The footprint of epistasis for a single pair in a long genome

The effect of epistasis on the evolution speed merits consideration but does not help to measure the interaction between genomic sites, which is the aim of our project. Therefore, we sought a footprint of epistasis *E* that would work for a single pair of sites and depend weakly on other sites and other model parameters. In this study, we will only consider the regime of negative selection, in particular, the case of weakly deleterious mutations, *s* < 0, |*s*| << 1 with positive epistasis, which here represents antagonistic epistasis, where the combined effect of two interacting alleles is less deleterious than the sum of their independent effects.

**Fig 2:**
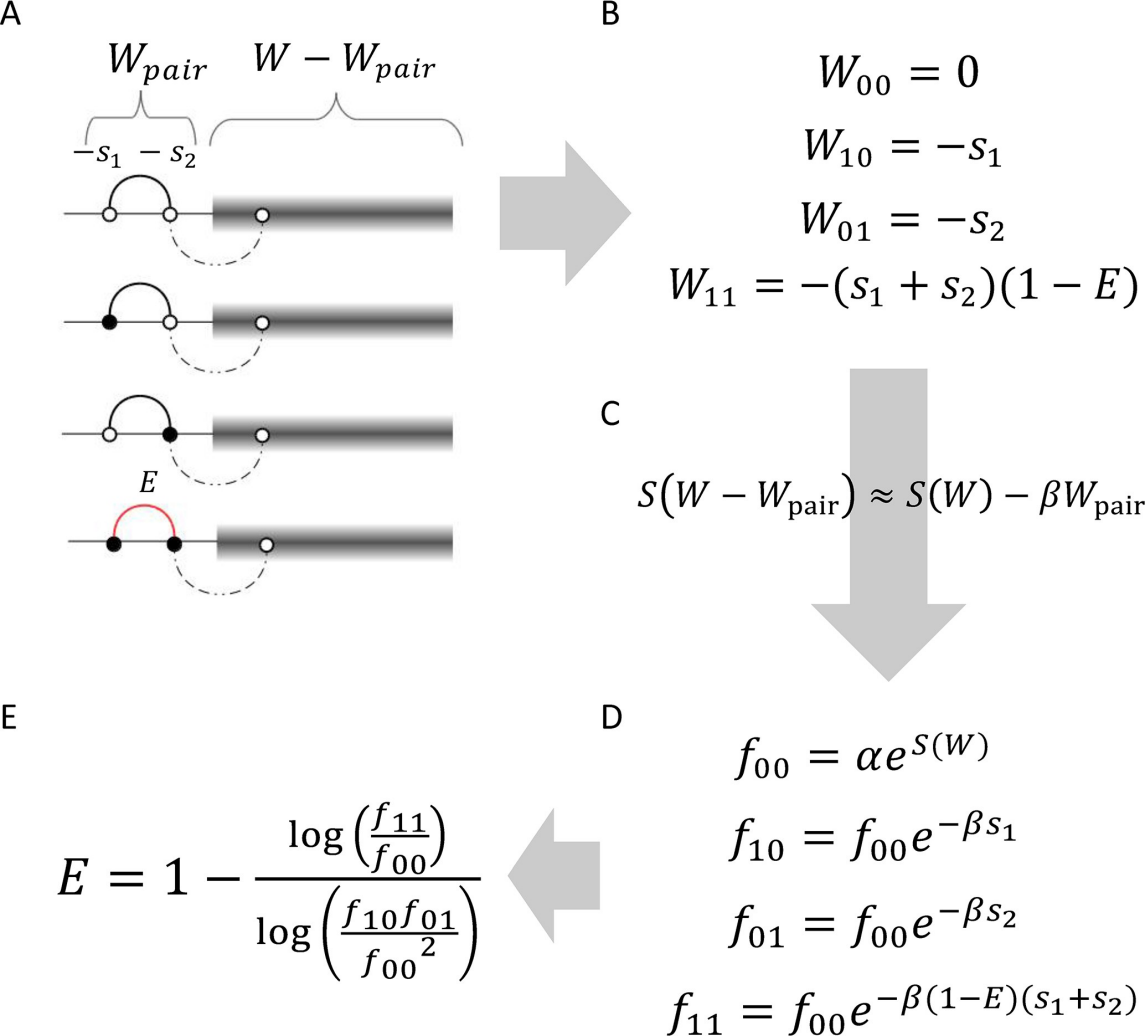
A pair of interacting sites in a long genome. (left) Open and filled circles: wild type 0 and mutated allele 1. Red line: existing interaction. Black line: potential interactions between sites. Dashed line: negligible interaction. Grey box: the rest of genome. (B-E) Derivation of the universal footprint of epistasis explained in the text. *W* is total fitness, *S*(*W*-*W*_pair_) is entropy of the rest of genome, and *f*_ii_ are the haplotype frequencies. Parameter *E* represents the relative strength of epistasis (Eq 1).

Consider an interacting pair of sites with selection coefficients –*s*_1_, −*s*_2_ and epistatic strength *E* (Eq 1) in a long genome with total log-fitness *W* (Fig 2, left panel). The population is assumed to be approaching mutation-selection equilibrium but not there yet. The models of population genetics demonstrate that the distribution density of fitness *W* across individual genomes is narrow for any reasonable population size met in experiments [49–52]. In the context of our work, we can consider it fixed at each given moment of time. The distribution in *W* represents a traveling wave which moves slowly towards higher *W*. Below we do not consider the wave’s shape explicitly but instead take advantage of the fact that the wave is narrow and slow.

We classify all individual genomes in the population into 4 groups, according to the haplotype sequence of the pair: 00, 01, 10 and 11. The fitness contribution of the pair, *W*_*pair*_, to the total genome fitness depends on the haplotype sequence (Fig 2).

$$W_{00}=0$$

$$W_{01}=-s_{1}$$

$$W_{10}=-s_{2}$$

$$W_{11}=-(s_{1}+s_{2})(1-E)$$ (3)

We assume that the epistatic pair does not interact with other mutated sites elsewhere in the genome. In other words, we neglect the existence of mutated clusters larger than two sites. In the following sections, we will lift this approximation and consider the effect of larger mutated clusters.

In the course of evolution, random drift and mutation tend to maximize disorder. On the other hand, the effect of Darwinian selection is to maximize fitness. The standard measure of disorder is configuration entropy *S* defined as the logarithm of the number of possible configurations, *S* = ln *N*_conf_. The compromise between the increase in entropy and the increase in fitness is satisfied when entropy is maximal under the restriction that fitness value *W* is fixed. As we mentioned above, different models of asexual evolution predict that the distribution in *W* is narrow and changes slowly. Hence, we make the hypothesis that entropy has enough time to nearly reach its maximum. At each moment of time, the maximum value of *S* depends on *W*, as given by *S* = *S* (*W*). Examples of function *S*(*W*) are considered in S1 Table (S1 Appendix).

Again, focus on a pair of sites (Fig 2). Consider all the sequences in the population, which have the same haplotype at the pair, for example, 10. We remind that the genome part not including the pair (grey box in Fig 2) is genetically diverse. The probability of appearance of haplotype, *f*_10_, by the definition of probability, is proportional to the number of possible sequence configurations *of the rest of genome* (grey box in Fig 2), exp(*S*_rest_). Entropy *S*_rest_ is restricted by fitness of the rest of genome, which is the difference *W*−*W*_pair_. Hence, we obtain that the entropy of each haplotype subset is *S*_rest_ *= S*(*W − W*_pair_). Further, since the genome is long, we can safely assume that *W*_pair_ is much smaller than *W*, so that the corresponding change in entropy is small and proportional to *W*_pair_. Hence, we can approximate

$$S(W-W_{pair})\approx S(W)-\beta W_{pair}$$ (4)

The frequency of each haplotype is proportional to the corresponding configuration number, exp[*S*(*W*−*W*_*pair*_)]. Combining Eqs 3 and 4, we can express the haplotype frequencies in terms of *s*_1_, *s*_2_, and *E*

$$f_{10}=f_{00}e^{-\beta s_{1}}$$

$$f_{01}=f_{00}e^{-\beta s_{2}}$$

$$f_{11}=f_{00}e^{-\beta {(s}_{1}+s_{2})(1-E)}$$ (5)

After excluding β, *s*_1_ and *s*_2_ from these expression (Eq 5), we arrive at the relationship between haplotype frequencies

$$\frac{f_{11}}{f_{00}}={\left(\frac{f_{10}f_{01}}{{f_{00}}^{2}}\right)}^{1-E}$$ (6)

Since haplotype frequencies can be measured, but epistatic strength is usually unknown, Eq 6 represents a "footprint of epistasis." It can be used to estimate the strength of interaction *E* in a single data set. Unlike the existing measures of linkage disequilibrium, the measure has direct biological meaning and a fair degree of universality. Henceforth we will refer to it as Universal Footprint of Epistasis (UFE).

Free bonus of this method is that the expressions for haplotype frequencies (Fig 2D) can be used to measure selection coefficients *s*_1_ and *s*_2_ from a diverse sequence set. Unknown parameter β is the same for all sites and can be found by averaging the frequencies over the genome.

**Fig 3:**
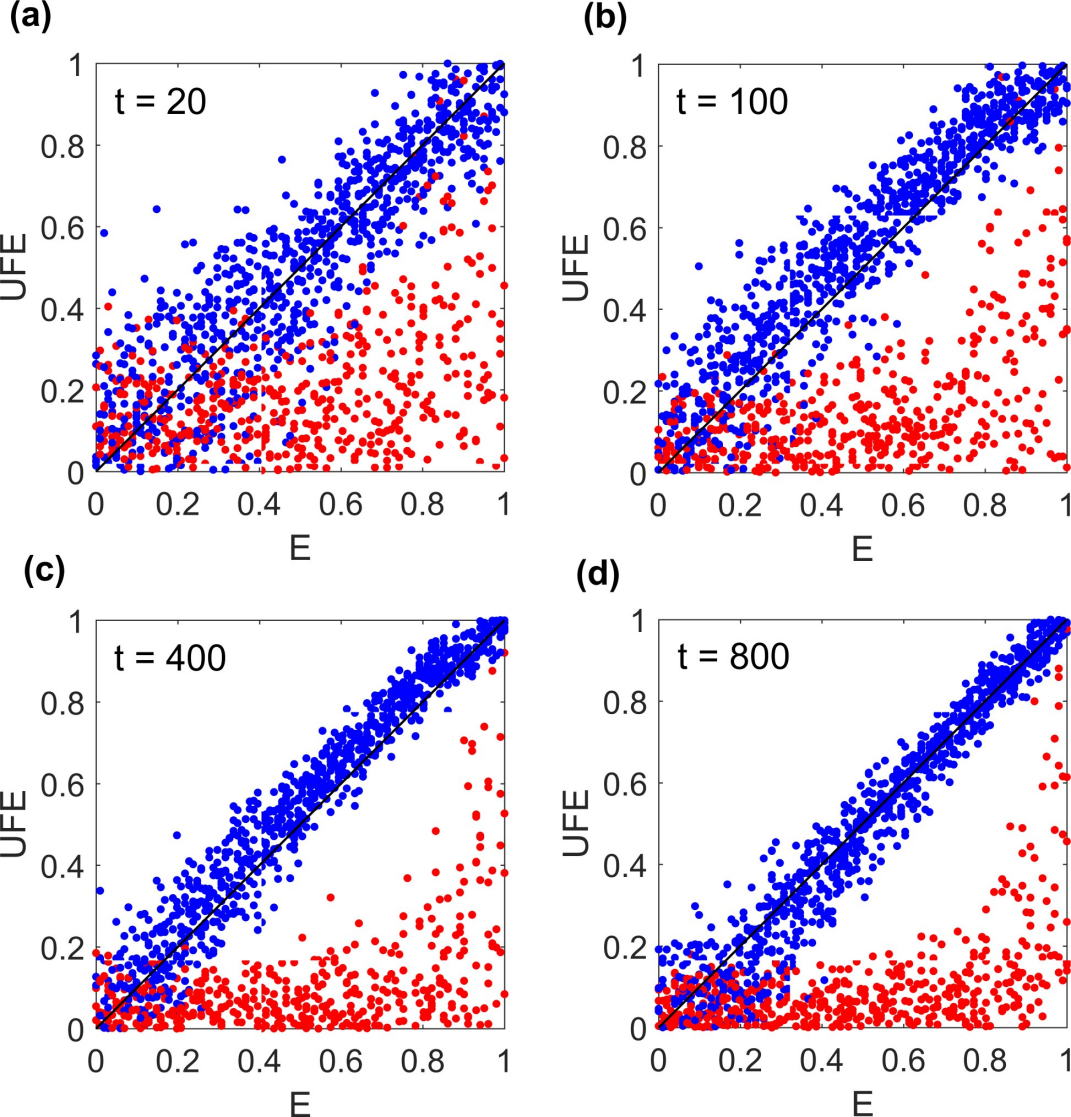
Universal footprint of epistasis (UFE) predicts epistatic strength in a broad time range. Value of *E* estimated from Eq 6 is plotted as a function of the actual value of *E*, where (a-d) correspond to different time points. Each dot represent a single Monte-Carlo run. Initial population is randomized with *f* = 0.5. Haplotype frequencies in Eq 6 are averaged over sites and pairs. Blue: known epistatic pairs. Red: the same number of randomly chosen pairs. Parameters: *L* = 300, *s*_0_ = 0.05, *N* = 500, μ*L* = 0.5, ~1 interaction per site.

We tested Eq 6 by Monte-Carlo simulation assuming isolated epistatic pairs and fixed *E*. The value of *E* was estimated from Eq 6 and compared with the actual value (Fig 3). Two parameter regions including the stochastic and quasi-deterministic regimes of evolution, which occur respectively at (*s*/μ*L*)log(*Ns*) < 1 and > 1, have been studied [49, 50]. The simulation shows that UFE estimate is established surprisingly early, after ~ 1/*s*_0_ generations, much earlier than the population arrives at equilibrium (Fig 3). We observed similar results at other parameter sets including much larger *N* (S2 Fig). However, after very long time, equilibrium is well established, and diversity becomes very small, *f* ~ μ/*s*. In this range, mutation balances selection and mixes different haplotypes. In this regime, deviations from UFE occur (S2 Fig).

### The long genome of isolated pairs

Above we considered a single pair in a genome. To further verify the validity of Eq 6, we will now consider the entire genome, for several examples of the interaction network. We start from the most straightforward "network" comprised of isolated pairs and assume that selection coefficient and epistatic strength are the same for all sites and pairs, *s*_i_ = -*s*_0_, *E*_*ij*_ = *E*_0_. This topology is relevant for genomes with sparse interacting sites. As we mentioned (*Methods*), the existence of non-interacting sites can be ignored. Examples of more complex topology will be considered in the next section.

**Fig 4:**
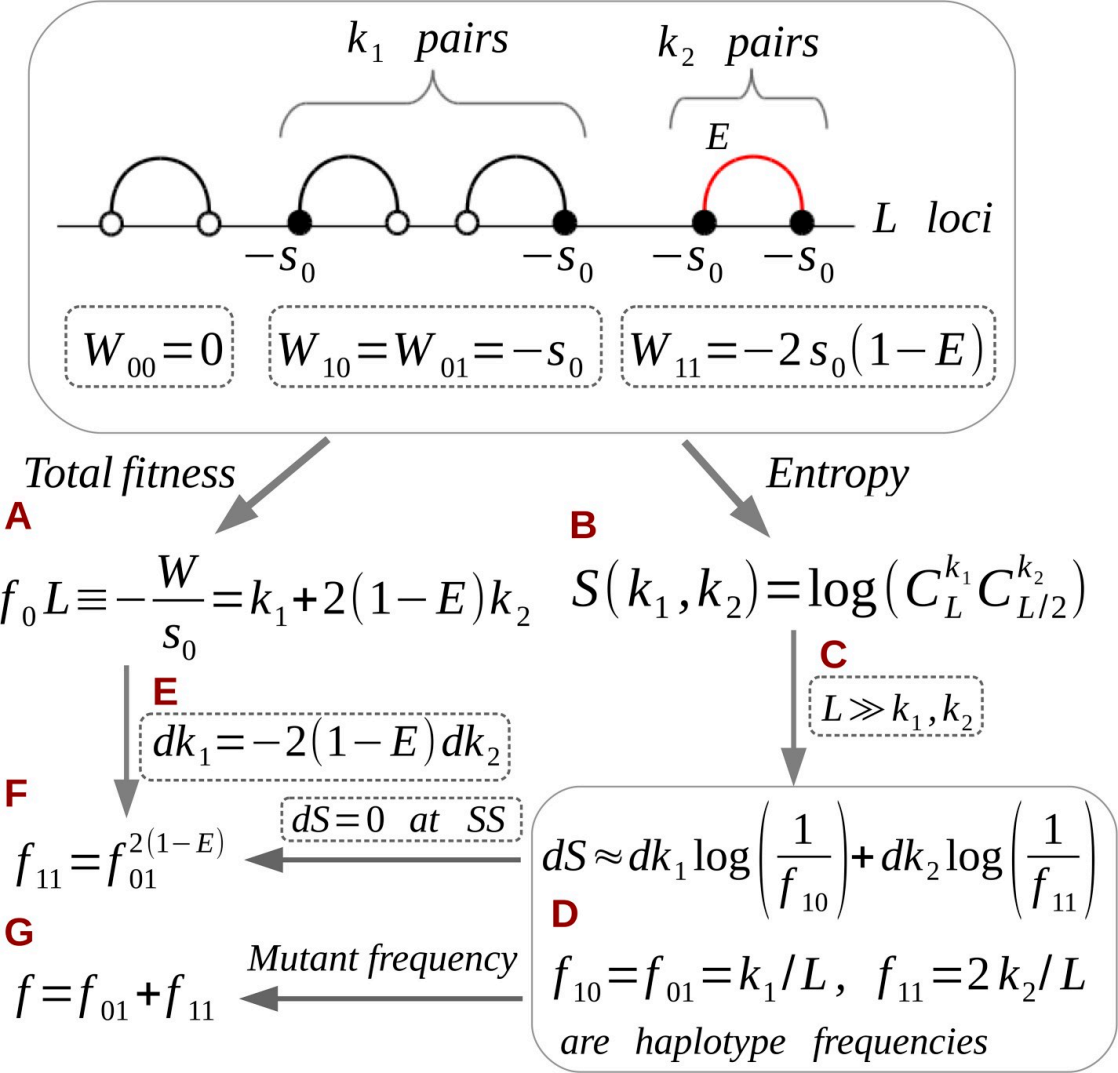
Long genome of interacting pairs. Top: Linked interacting pairs with different haplotypes and their fitness values *W*_*ij*_. (a—g) Flow chart of the derivation of the universal footprint of epistasis (see the text or S1 Appendix).

First, we group mutated clusters by their size and monitored the group numbers: *k*_1_ single mutations and *k*_2_ connected mutated pairs (Fig 4, top). The fitness number and entropy can both be expressed in term of the numbers of singles and doubles (Fig 4A and 4B). In the most probable state of the system, the values of *k*_1_ and *k*_2_ are chosen to maximize entropy *S* under the restriction that fitness *W* is fixed. Assuming that mutations are rare in the genome (Fig 4C), we can approximate *S* by a continuous function of *k*_1_ and *k*_2_ and find its derivatives in these variables (Fig 4D). Next, the average frequencies of haplotypes 10 and 00 can be expressed regarding *k*_1_ and *k*_2_ (Fig 4D). Finally, from the condition that entropy is maximum and the condition that fitness number is fixed (Fig 4E), we arrive at a relation between haplotype frequencies (Fig 4F). The relationship is identical to UFE, Eq 6, when mutations are rare, *f*_00_ ≈ 1. To express *f*_10_ and *f*_11_ in terms of mutation frequency *f*, we use the condition that their sum is equal to *f* (Fig 4G).

From Eq 6, at half-compensation point *E* = 0.5, mutated pairs and singles have the same frequency *f*_11_ = *f*_10_, because they have the same mutation cost (Fig 4A). But slightly off this point, one group strongly outnumbers another: at *E* < 0.5, the singles are much more numerous, and the doubles dominate at *E* > 0.5. Thus, the presence of epistasis violates the common sense that double deleterious mutations are always more rare than single mutations.

**Fig 5:**
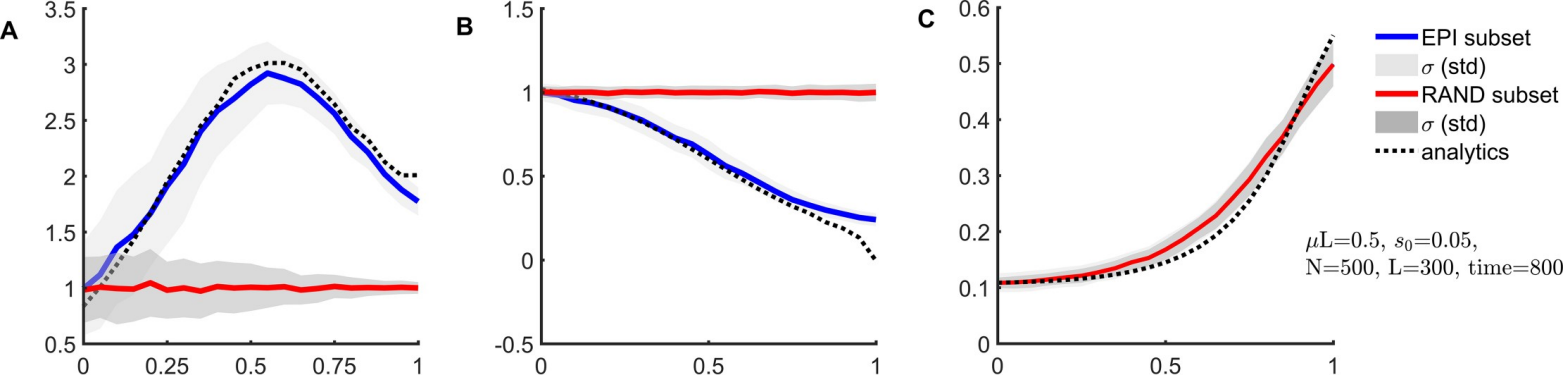
Epistasis causes strong linkage disequilibrium: Analytics and simulation. (a, b, c) Correlation coefficients *D*_11_, *D*_10_ and mutation frequency *f* are shown as a function of *E*. *D*_11_, *D*_10_, are calculated from Eq 6 using simulated values of *f*_*ij*_ and *f* averaged over sites and pairs. Color lines correspond to the average over 300 runs, and the shaded areas show the standard deviation among runs, for epistatic pairs (blue) and the same number of random pairs (red). Dotted black line is the analytic prediction. Parameters: *N* = 500, *s*_0_ = 0.05, *L* = 300, μ*L* = 0.5, *t* = 800, *f*_0_ ≅ 0.1. Initial population is randomized with *f* = 0.5. Thus, simulation agrees well with analytic predictions.

**Fig 6:**
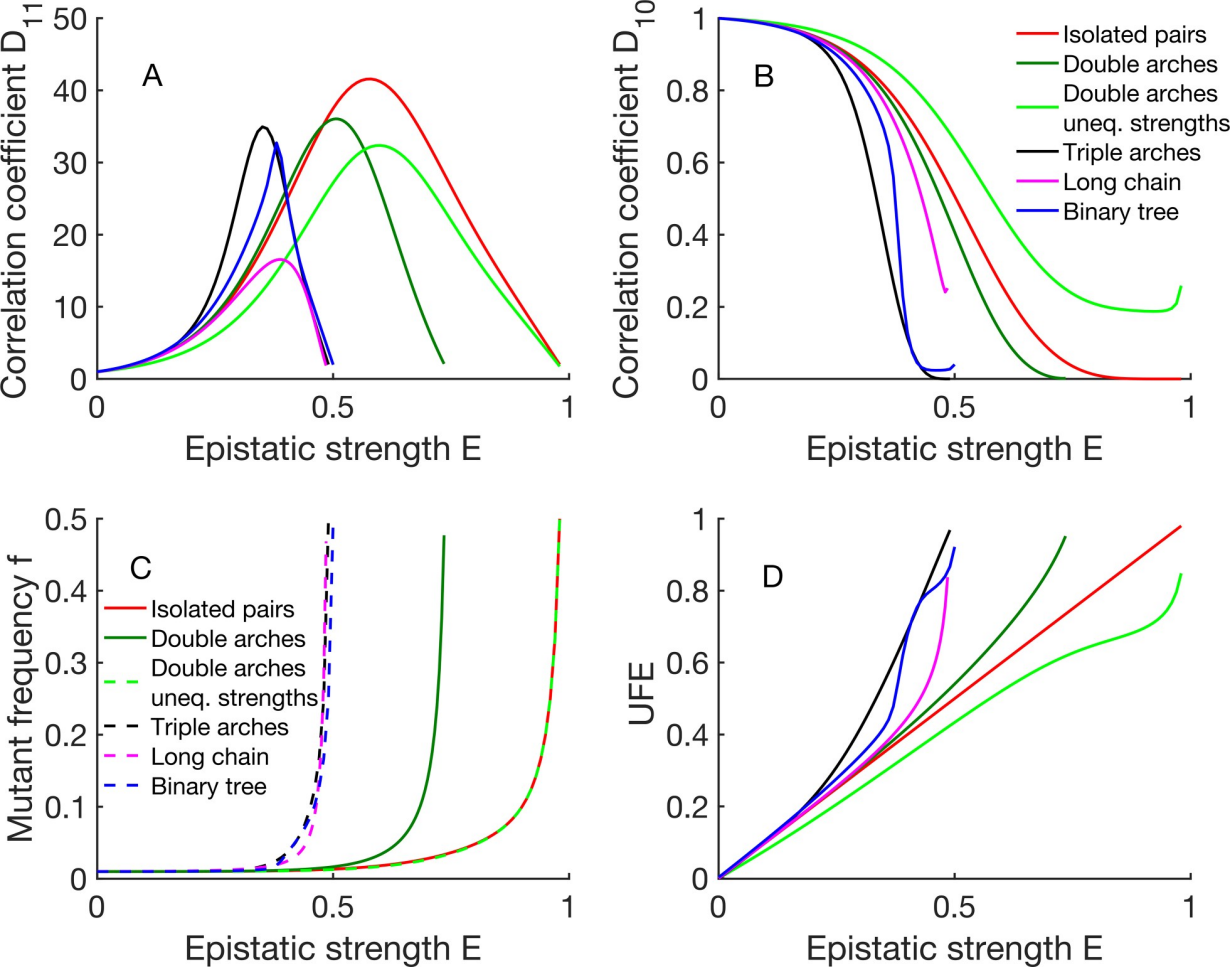
UFE is preserved for different topologies at moderate epistasis' strengths. Here we show the dependences on *E* for (a, b) correlation coefficients, (c) mutation frequency *f*, and (d) UFE on *E* for the five topologies in Fig 6. UFE is the estimate of *E* (Eq 6) from haplotype frequencies *f*_01_, *f*_11_ derived analytically for each topology (S1 Appendix). Parameters: *f*_0_ = 1/100. Thus UFE is exact for the isolated pairs, and overestimates *E* at large *E* for other topologies. Asymptotic expressions are given in S2 Table.

We also derive Lewontin's measure of linkage disequilibrium, correlation coefficients *D*_11_ = *f*_11_/*f*^2^, *D*_10_ = *f*_10_/[*f*(1 − *f*)]. With the use of Eq 6, we can express them in terms of mutant frequency *f* (S2 Table). In turn, mutant frequency *f* can be expressed in terms of input parameters *E* and *f*_0_. The results are compared with simulation for initial frequency *f*_0_ = 1/10 (Fig 5). The analytic results for smaller diversity, *f*_0_ = 1/100, are shown in Fig 6. As expected, mutant frequency *f* diverges near full compensation point *E* = 1 until reaches the value of 0.5 (Figs 5C and 6C red, S2 Table). This value follows directly from the symmetry between wild-type and mutant alleles existing at *E* = 1. Correlation coefficient *D*_11_ increases with *E*, peaks at *E* = 1/2, and then decreases linearly with *E* to the value of 2 until full compensation (Figs 5A and 6A red). Indeed, at that point we have *f*_00_ = *f*_11_ = 1/2 from the symmetry. In contrast, coefficient *D*_10_ stays near 1 and declines rapidly at *E* > 1/2 where the doubles outcompete the singles (Figs 5B and 6B red) until hits zero.

Most importantly, UFE relationship is exact in the entire interval of *E* (Fig 6D red and Fig 3). In what follows, we will consider UFE for more complex networks interaction.

### Full compensation and UFE interval

**Fig 7:**
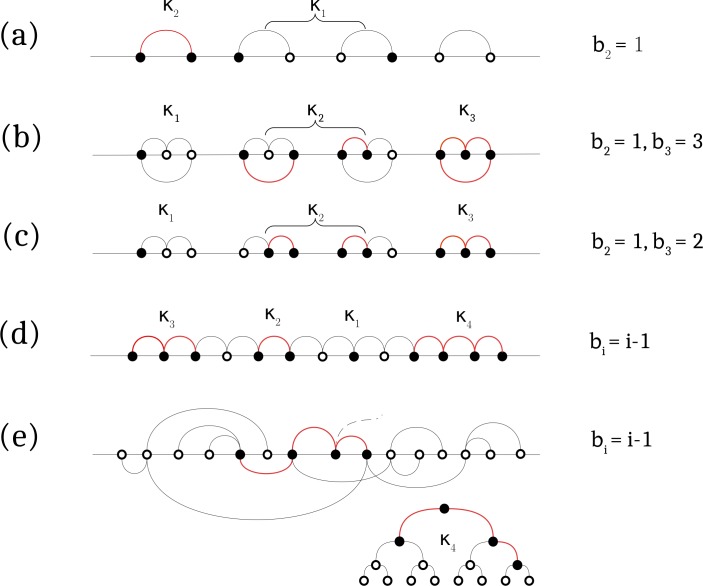
Examples of epistatic network. Filled and open circles denote mutated and wild-type genomic sites, respectively. Epistatic interactions between sites are shown by black and red lines. Red lines show clusters of mutated sites. *k*_i_ is the number of the cluster with *i* sites, *b*_i_ is the bond number per cluster. Different topologies correspond to a) isolated pairs, b) isolated triple arches, where each site has two epistatic partners, c) double arches, where three sites are involved in two epistatic associations, d) long connected chain where each site forms two pairs, (a-d) show connection topology but not the actual site order. (e) Binary tree: possible site order and the equivalent tree structure.

The topology of actual epistatic interactions can be more complex than isolated pairs (Fig 7B–7E). In the following section, we will study specific examples. However, even before, we can obtain two general results for any topology based on the general expression for fitness (*Methods*, Eq 9). The first observation is that when epistatic strength *E* exceeds a critical value *E*_*c*_ given by

$$E_{c}={min}_{i}[i/\left(2b_{i})\right]$$ (7)

where *b*_i_ is defined as the number of bonds in a cluster of size *i*, some mutated clusters become over-compensated. The point *E*_*c*_ represents the threshold of full compensation for a cluster, which means the loss of genetic stability for the entire population (S2 Table). Above *E*_*c*_, the critical clusters will rapidly expand in time until the entire genome has mutated. If mutations have an unwanted phenotype, such as drug resistance or cancerogenic potential, this is the point where a virus becomes resistant, or a tumor starts to grow (we do not address the immune system effects).

We remind that, for isolated pairs, the point of full compensation is *E*_c_ = 1. In the general case, the value of compensation point *E*_*c*_ (Eq 7) is either equal or less than 1. The exact value depends on topology, i.e., on the number of bonds *b*_*i*_ (Fig 7). The reason why *E*_c_ can be less than 1, is that each mutation can compensate more than one mutation.

The second general result is that the UFE estimate of *E* (Eq 6) is predicted to be accurate at *E* < *E*_*UFE*_, where

$$E_{UFE}={min}_{i>2}\left[\frac{i-2}{2(b_{i}-1)}\right]\leq 1/2$$ (8)

represents the point where an interacting cluster larger than two sites is as fit as two interacting sites (S2 Table). Beyond *E*_*UFE*_, the doubles are outnumbered by larger clusters. As a result, the predicted value of UFE overshoots the value of *E* in Eq 6 that was derived by taking into account only isolated pairs (Fig 6D). In other words, the value of *E*_*UFE*_ defines the interval within which UFE in Eq 6 is accurate. As we see from Eqs 7 and 8, topology of network affects both *E*_*c*_ and *E*_*UFE*_ through the number of bonds *b*_i_ for cluster of each size *i*.

### Effects of network topology

In the previous sections, we ignored the clusters of more than two interacting mutated sites because we considered isolated interacting pairs (Fig 7A). For more complex network topology, triplets and larger clusters may become important. In this section, we consider several examples (Fig 7B–7E). We follow the general algorithm, as follows. The derivation for each topology starts from the expressions for entropy, fitness, and haplotype frequencies as the numbers of mutated interacting clusters of different size (section *Methods* below). Detailed derivations are given in *S1 Appendix*. The starting analytic expressions and analytic results are listed in S1 and S2 Tables, respectively (S1 *Appendix*). Here we only discuss final results qualitatively.

#### Triple arches

Consider the periodic sequence of three-node graphs connected by three bonds (Fig 7B). In this network, triple interacting mutations are possible. Now entropy and fitness depend on three variables: number of single mutations, *k*_1_, of the doubles, *k*_2_, and of the triplets, *k*_3_ (S1 Table). As in the case of isolated pairs we have considered in the previous section, we maximize entropy in these three variables while keeping fitness fixed. With this topology of interactions, the fitness loss of a triple arch structure, which consists of three deleterious alleles linked by three epistatic interactions, is fully compensated when *E* = 0.5. Indeed, the existence of extra interactions lowers the point of full compensation *E*_*c*_, which in this case, decreases from 1 to 1/2 (Fig 6A and 6B; S2 Table). Correlations coefficients *D*_10_, *D*_11_ vary with epistasis strength in a different way within three intervals of *E*, as follows: At *E* below 1/4, there are few triplets compared to the doubles, i.e., *k*_3_ << *k*_2_. In this interval, the universal relationship, Eq 6, is accurate (Fig 6D). For more substantial epistatic strengths, 1/4 < *E* < 1/3, the triplets outnumber the doubles, *k*_3_ >> *k*_2,_ and linked pairs of mutations are found mainly in triplets. As a result, UFE is modified: at *E* > 1/4, correlations are stronger, and UFE estimate (Eq 6) predicts a larger value of *E* than the actual value (Fig 6D), which is also reflected in a steeper increase of *D*_11_ with *E* than for the case of isolated pairs. At an even higher degree of compensation, 1/3 < *E* < 1/2, triplets outnumber even single mutations. Approaching the point of full compensation *E* = 1/2, the accumulation of triplets causes divergence of *f* and a linear decrease in *D*_11_ (Fig 6C and 6D). Thus, extra bonds between interacting sites generate a positive correction to UFE.

#### Double arches

To test this conclusion further, we removed a bond from each triple arch (Fig 7C). The changes from the previous case are shown in Fig 6, and S2 Table. The intermediate interval of *E*, 1/4 < *E* < 1/3 disappears, because the singles, doubles, and triplets have the same fitness and, hence, similar abundance at the same point, *E* = 1/2. The single mutations are most numerous below this point, and the triplets dominate above. Further, the interval of UFE validity expands from *E*_UFE_ = 1/4 to *E*_UFE_ = 1/2, and full compensation occurs at larger *E*, *E*_*c*_ = 3/4.

#### Chain

Next, we consider a long chain of interacting adjacent sites (Fig 7D). In this topology, mutated clusters of any size *i* smaller than a total number of sites *L* can exist, with *b*_*i*_ *= i—*1 epistatic bonds. Which size of clusters is the most important? After maximizing entropy at fixed fitness, we obtain that the frequencies of clusters of different size form a geometric progression (S1 Appendix). Due to the assumption of small mutant frequency *f*, we obtain that the denominator of the progression is very small, unless very close to the full compensation point *E*_c_ < 1/2. Hence, at most values of *E*, we can neglect clusters larger than *i* = 2, which again produces the universal result UFE (Fig 6D, S2 Table). Thus, for the extended chain topology, UFE formula is valid in the most of the interval of *E*. Only in a narrow vicinity of compensation point, large clusters become important causing divergence of *f* and overestimation of E from UFE (Fig 6D).

#### Binary tree

A tree is a graph without loops where any two nodes can be connected by a single path (Fig 7E). Analysis for the binary tree and the chain is similar. The relationship between the number of bonds and nodes stays the same, *b*_*i*_ = *i*-1, and so does the critical point *E*_c_ = 1/2. The difference is in entropy: each mutated cluster of size *i* is now a subtree that has *n*_i_ = (2*i*)!/[*i*!(*i*+1)!] possible shapes. Instead of only one as in the previous case, which fact favors larger clusters (even though their fitness stays the same as in the chain). Consequently, once larger clusters become essential near *E*_c_, correlation coefficients and UFE increase rather sharply, and the peak in *D*_11_ is taller than for the chain (Fig 6A). UFE dependence applies in most of the interval until close to the compensation point (Fig 6D). The same qualitative conclusions hold true for a tree with any, even random number of branches.

#### Non-epistatic sites

For our aim, the existence of non-interacting sites in the genome can be just ignored (S1A Fig), because fitness *W* and entropy *S* are additive over epistatic and non-epistatic part, and the frequencies *f*_11_, *f*_10_, *f*, and *f*_0_ can be defined for epistatic sites only. Hence, one can maximize the entropy of the epistatic part given its fitness independently of the non-epistatic part. For the same reason, a diverse mixture of different graphs can be split into uniform segments (S1A Fig). Each segment can be treated separately, as we described above, then, the total entropy of the combination of segments can be maximized.

#### Doubles arches with unequal interactions

So far, we considered different topologies with equal epistatic strengths of interacting pairs. Because in real genomes epistasis strength varies, here we provide a sensitivity test in the case of "double arches" (Fig 7C). Here we assume that the left bond of each double arch has epistatic strength *E* and the right bond has strength *E/*2. For a detailed derivation of the correlation coefficients and UFE in various intervals of *E*, see S1 Appendix, Section 3.6. The results are summarized in S2 Table. In S3 Fig, we compare the results for *D*_11_, *D*_10_, *f* and UFE relation between the cases of equal and unequal interaction.

In contrast to the case of equal interactions, we obtain three intervals of *E* instead of two (see S2 Table). Full compensation occurs later, *E*_c_ = 1, as in the case of isolated pairs (see S2 Table). Interestingly, correlation coefficient *D*_10_ in the last interval (2/3,1) changes its behavior qualitatively (see S3 Fig and S2 Table). In the case of equal epistatic strength, it decreased exponentially in the last interval of *E*. For unequal epistatic strength, it decreases more slowly, as a power law. The UFE relation now does not overshoot the value of *E*, but rather slightly underestimates it (see S3 Fig), albeit it remains close to *E* in the whole interval of *E*. This is because UFE represents an intermediate value between the two values of *E*. Yet, UFE is closer to the larger of the two.

## Discussion

Using analysis and Monte-Carlo simulation of adapting asexual population, we obtained a relationship between haplotype frequencies of a pair of sites *f*_11_, *f*_10_, and *f*_00_, which can serve to measure the strength of pairwise interaction *E* (UFE). At moderate epistatic strengths *E*, the relationship of UFE is shown to be independent on the topology of the epistatic network, and any system parameters other than *E*. For example, selection coefficients, mutation rate and population size may be unknown, which fact does not affect the results. For the simplest topology of isolated epistatic pairs, this result applies in the entire interval of *E*. For more complex networks and stronger interaction; we predict a transition to the case where the haplotype relationship acquires topology-dependent correction and may overestimate *E* by a factor less than 2. We showed that the point of full compensation in *E* and the interval where UFE is accurate decrease with the number of interactions per interacting site. We can use this information for a biomedical purpose, such as identifying the clusters of compensatory mutations critical for the evolution of drug resistance [38, 39, 42–44].

Our results demonstrate the existence at any time point of a quasi-equilibrium between Darwinian fitness and disorder (entropy) due to random genetic drift and mutation. The reason is a relatively slow rate of asexual adaptation due to clonal interference effects leading to the formation of a traveling wave with slowly changing parameters [51, 52, 53, 60]. Hence, UFE has time to form within fitness classes while the wave is slowly traveling.

The present analysis has limitations, as follows:

- To use UFE as a measure of the strength of epistasis, one needs a population with a sufficiently high initial variation.
- On very long times, significant deviations from UFE occur that progressively wipe away the epistatic footprint at *E* < 1/2 (S2 Fig). The reason is a mutation which, although helping genetic drift create disorder, also mixes the haplotypes between sequences, thus smearing UFE. The last effect becomes strong when the mutation-selection balance is established (S2 Fig).
- We averaged haplotype frequencies over epistatic pairs, which fluctuate among pairs and in time due to the stochastic nature of the system. Our next step will be to include the statistical inference from real sequence data to the method.
- We considered asexual haploid populations. Sufficiently strong recombination can mask epistasis. For example, if a virus variant can recombine well, and another variant cannot recombine as much, the comparison of how the UFE model fits the data of each virus would allow us to measure the effect of recombination on epistasis.

To summarize, we propose an analytic tool to measure epistatic interaction in natural selection. Detection noise and the generalization of our results to sexual populations will be addressed elsewhere.

## Methods

### Fitness for uniform selection parameters s and E

We consider a weakly diverse population near equilibrium and assume that all mutations are deleterious with equal selection coefficient *s*_*i*_ = -*s*_0_ < 0, and that epistatic strength is fixed as well, *E*_*ij*_ = *E* > 0. In this case, we can characterize a genome by the numbers of mutated clusters of different size. To do so, let *k*_*i*_ define the number of clusters with *i* nodes and *b*_i_ bonds (Fig 7). Generally, *b*_i_ can take multiple values for each cluster size *i* (S2 Fig). For the sake of simplicity, we will consider topologies in which *b*_i_ assumes a single value for each cluster size *i* (Fig 7). Then, from Eq 1, we can express fitness as a sum over clusters of different size

$$W{\equiv -s}_{0}f_{0}L={-s}_{0}\sum_{i=1}^{i_{max}}k_{i}(i-2Eb_{i})$$ (9)

New notation *f*_0_ represents the frequency of uncompensated mutations with total fitness *W*. The number of bonds *b*_i_ for cluster size *i* > 2 depends on the topology (Fig 7), but for single and double mutations, we always have *b*_1_ = 0, *b*_2_ = 1. To avoid possible confusion, Fig 7 represents topologies of the network regardless of the actual location of the sites in the genome.

### Quasi-equilibrium state: Entropy

As we demonstrate by simulation, at each moment of time, *k*_i_ are determined by the condition that the entropy of the system is maximum given the value of fitness (Eq 9). Entropy *S* is defined as the log number of configurations

$$e^{S}=\prod_{i=1}^{i_{max}}C_{L_{i}}^{k_{i}}{\left(n_{i}\right)}^{k_{i}}$$ (10)

where *L*_i_ is the number of all possible locations for a cluster of size *i*, and *n*_i_ is the number of each cluster's configurations (shapes). The values of *L*_i_ and *n*_i_ depend on network's topology. In Eq 10, we neglect the overlap between clusters of different size due to the condition that mutations are sparse. (The condition does not hold in the vicinity of full compensation, *E* = *E*_c_, where *f* sharply increases). In what follows, we consider maximum entropy concerning the values of *k*_i_ with the fitness restriction (Eq 9).

### Pairwise mutation frequencies (haplotypes)

Through most of the manuscript, we assume that the one-site frequency of deleterious mutations

$$f=\frac{1}{L}\sum_{i}{ik}_{i}\;<<\;1$$ (11)

is small. From Eq 9, *f*(*E* = 0) = *f*_0_ = *k*_0_/*L* which represents the "negative fitness density" per site. The value of *f*_0_ may depend on the state of the population and system parameters. At equilibrium, the dependence of *f*_0_ on *E* in steady state is also relatively slow: between *E* = 0 and 1, only 2-fold [49, 50]. To avoid these complications and focus on strong effects of epistasis, we treat *f*_0_ as an input parameter and assume that it changes in time and *E* slowly. The distribution of genomes in fitness is narrow in a broad range of parameters and times scales [49], and we assume *f*_0_ to be the same for all genomes in the population. The dependence *f*(*E*)/ *f*_0_ can be derived from Eq 9. At positive *E*, we have *f*(*E*) > *f*_0_.

To obtain a footprint of epistasis, we need to express haplotype frequencies in terms of numbers of clusters of various size, *k*_i._ We calculate the frequencies of haplotypes 00 and 01

$$f_{11}=\frac{1}{L_{pair}}\sum_{i}k_{i}b_{i}$$

$$f_{10}=f_{01}=f-f_{11}$$ (12)

where *L*_*pair*_ = ∑_*ij*_*T*_*ij*_ is the total number of interacting pairs in the genome, and the correlation coefficients *D*_*ij*_

$$D_{11}=\frac{f_{11}}{f^{2}},\;D_{10}=\frac{f_{10}}{f(1-f)},$$ (13)

If two sites are statistically independent, by definition, *D*_11_ = *D*_10_ = 1.

## Supporting information

S1 Appendix (PDF)

S1 Table Expressions for fitness -*W*/*s*_0_, the exponential of entropy *e*^S(k_1_,k_2_)^, and haplotype frequencies *f*_10_, *f*_11_ in different cases of epistatic network topology. Note that in the expression for exponential of entropy we neglect *k*_i_ compared to *L*.
(PDF)

S2 Table Correlation coefficients and critical points for five examples of network topology. See Fig 6, main text. Here *b*_i_ is the number of bonds for a cluster of *i* mutations, *E*_c_ is the point of full compensation, *E*_UFE_ is the maximum epistatic strength at which UFE still applies. *D*_11_ and *D*_10_ are pairwise correlation coefficients, *f* is mutant allelic frequency.
(PDF)

S1 Fig Variations of topology. (a) A diverse topology consisting of finite sub-graphs of epistatic interactions can be considered as a composition of uniform topologies (i.e. single pairs, double and triple arches), setting aside non-epistatic loci. (b) Example of the complex topology, when two sub-clusters of the same size may have different number of bonds (not considered in this work).
(TIFF)

S2 Fig The value of *E* estimated from UFE relation (Eq 6 or Eq 2.2) as a function of the actual value of *E*. Shown are times in generations. Bottom right: Time evolution of the mutant frequency *f*. Each dot represents a single Monte-Carlo run. Initial population is randomized with *f* = 0.5. Haplotype frequencies in Eq (2.2) are averaged over sites and pairs. Blue: known epistatic pairs. Red: the same number of randomly chosen pairs. Parameter set: *L* = 100, *s*_0_ = 0.05, *N* = 2000, *μL* = 0.2, one bond per interacting site.
(TIFF)

S3 Fig Effect of unequal epistatic strength for double arches. Shown are correlation coefficients, average frequency and UFE as a function of *E* in the case of equal epistatic strength (green) and in the case of unequal strengths *E* and *E*/2 (blue), *f*_0_ = 1/100 is input parameter.
(TIFF)
