# Supplementary material for: Evolutionary footprint of epistasis
Source: PLoS Comput Biol. 2018 Sep 17;14(9):e1006426. doi: 10.1371/journal.pcbi.1006426 (PMC6177197; doi:10.1371/journal.pcbi.1006426)
Supplement: S1 Appendix — (PDF) [file pcbi.1006426.s001.pdf]

Supplemental information: mathematical appendix

Evolutionary footprint of epistasis

G. Pedruzzi, A. Barlukova, I.M. Rouzine

Sorbonne Université, Institute de Biologie Paris-Seine, Laboratoire de Biologie Computationelle et Quantitative, LCQB, F-75004 Paris, France

Contents

1 Model of pairwise interaction **1**

2 General case of one pair of sites **2**

3 Different topologies of epistasis **3**

3.1 Isolated pairs 4

3.2 Double arches 7

3.3 Triple arches 10

3.4 Connected long chain 13

3.5 Large binary tree 17

3.6 Double arches with unequal interactions 20

4 Supplementary references **25**

1 Model of pairwise interaction

We consider N binary sequences of L loci long. Each allele K_i, $i = 1, \dots, L$, is either equal to 0 for wild-type, or 1 if locus i is mutated. Darwinian fitness of a genome is defined as the logarithm of the average progeny number of a sequence. We assume it to have the form

$$W = \sum_{i=1}^{L} s_i K_i + \sum_{\substack{i=1 \\ j<i}}^{L} S_{ij} K_i K_j \quad \text{where} \qquad (1.1)$$
$$S_{ij} = E_{ij}(|s_i| + |s_j|)T_{ij}$$

Here, $s_i \ll 1$, $i = 1, \dots, L$, are selection coefficients. The first term in (1.1) stands for the effect of single sites on fitness, while the second one describes the effect of pairwise interactions due to epistasis. The term T_{ij} is equal to 1 for any pair of loci i, j linked by epistasis, and 0 for non-linked sites. Epistatic

coefficient E_{ij} represents the relative strength of pairwise interaction. In this work, we focus our attention on the case of negative selection coefficients (i.e. mutated alleles are less fit than wild-type ones), and positive epistasis $E_{ij} > 0$. We also assume $E_{ij} < 1$ to avoid the case of over-compensation, which can be reduced to under-compensation by symmetry transformation $K_i = 1 - K_i$.

2 General case of one pair of sites

Consider now a weakly diverse population of genomes and focus on two locus positions that are linked by epistasis. Without loss of generality, we can denote the loci of interest as "1" and "2" (see Fig. 2 in the main text). Let s_1 and s_2, be the selection coefficients for the chosen sites, E be the coefficient for epistatic strength, and W the total fitness. According to (1.1), there are three cases for the contribution of the epistatic pair W_{pair} to total fitness W:

$$\begin{aligned} W_{pair} &= -s_1 && \text{if the first site of the pair is mutated} \\ W_{pair} &= -s_2 && \text{if the second site of the pair is mutated} \\ W_{pair} &= -(1-E)(s_1+s_2) && \text{if both sites of the pair are mutated.} \end{aligned}$$

Since the probability density function of fitness in real genomes is narrow [51] (see Methods in the main text), we assume that the genome is sufficiently long to consider W as a fixed value. Then, for the rest of the genome, the fitness is $W - W_{pair}$.

We denote the entropy of the system by S, which is the logarithm of the number of all possible configurations of the genome

$$S = \ln(N_{conf})$$

Note that N_{conf} depends on the topology of the sites network and in the following section, we investigate several particular cases of topology in detail (please see Table S1). We assume a population at the maximum degree of disorder which corresponds to maximal entropy. However, any given sequence is also under the restriction of that fitness is fixed. Therefore, the maximum entropy behaves as a function of fitness, $S(W)$. At this point, an explicit expression for $S(W)$ is not required. In the following section, we describe a procedure of entropy maximization in some special cases of topology of interaction network.

Small change in the argument $W - W_{pair}$ of S can be approximated as

$$S(W - W_{pair}) \approx S(W) - W_{pair} S'(W) \tag{2.1}$$

We denote $S'(W) \equiv \beta$.

Let f_{00}, f_{01}, f_{10}, f_{11} be the frequencies of pairs with corresponding haplotypes 00, 01, 10 and 11. The frequency f_{00} is proportional to the number of possible configurations $f_{00} = \alpha e^{S(W)}$, where $\alpha < 1$ is a constant. Then, using the formula (2.1), the other frequencies can be approximated by the formulae

$$\begin{aligned} f_{10} &= f_{00} e^{-\beta s_1} \\ f_{01} &= f_{00} e^{-\beta s_2} \\ f_{11} &= f_{00} e^{-\beta(1-E)(s_1+s_2)} \end{aligned}$$

where $f_{00} + f_{01} + f_{10} + f_{11} = 1$. Combining the equations in the following way:

$$\begin{aligned} f_{00} f_{11} &= f_{00}^2 e^{-\beta(1-E)(s_1+s_2)} \\ f_{01} f_{10} &= f_{00}^2 e^{-\beta(s_1+s_2)} \end{aligned}$$

we obtain the relation (Eq. 3, main text)

$$\frac{f_{11}}{f_{00}} = \left(\frac{f_{01} f_{10}}{f_{00}^2} \right)^{1-E} \tag{2.2}$$

Formula (2.2) is fairly general, as it does not depend on the genome parameters. However, notice that it has been obtained under some limitations. Here, we assumed that the epistasis for any links between the pair and the rest of the genome was rather weak. It allowed us to consider any epistatic pair separately. In the next section, we consider more complicated topology, where this assumption is lifted.

3 Different topologies of epistasis

From now on, we set

$$\begin{aligned} s_i = -s_0, \quad s_0 > 0, \quad i = 1, \ldots, L \\ E_{ij} = E, \quad i = 1, \ldots, L, \quad j < i \end{aligned} \tag{3.1}$$

All selection coefficients are negative, which means that all the mutations are deleterious. All the selection coefficients are equal, and so are the coefficients of epistatic strength. Under these conditions, below we consider the following topologies (Fig. 6 in the main text):

- Isolated pairs (Fig. 6a)
- Double arches (Fig. 6c)
- Triple arches (Fig. 6b)
- Connected long chain (Fig. 6d)
- Large binary tree (Fig. 6e)

By the word "isolated", we imply that the interactions between each pair with the rest of the genome are negligible. We also assume that

$$W \text{ is a fixed value}$$
$$0 < E < 1 \quad (3.2)$$
$$1 \ll k_i \ll L, \quad i = 1, \dots, I \quad (3.3)$$

where k_i is a number of clusters of i mutated sites linked by epistasis, I is the maximal possible cluster size for a given topology.

3.1 Isolated pairs

In Section 2, we studied a weakly diverse population of sequences with a focus on two chosen loci positions with known selection and epistatic coefficients, while the remaining parts of the genome was treated as a "black box". In the present section, we consider the entire sequence. All sites have the same selection coefficient and interact only as isolated pairs (see Fig. 6a, main text). Epistatic coefficient for all pairs is also the same. The aim of considering such a simplistic topology is to demonstrate the validity of universal result given by (2.2), which derivation may seem to be not quite intuitive.

We assume that entropy is at its maximum under the restriction of total fitness W given by Eq. (1.1), which, in the case of isolated pairs, can be written in the form

$$k_0 \equiv -\frac{W}{s_0} = k_1 + 2k_2(1 - E) \quad (3.4)$$

Here, for the sake of convenience, we introduce the notation $k_0 \equiv W/s_0$, where k_0 is constant; k_1, k_2 are the numbers of pairs with one and two mutated sites, respectively.

The entropy of the system S at given k_1 and k_2 is the logarithm of the number of possible configurations

$$e^S = N_{conf}$$

which, in our case, can be calculated as

$$N_{conf}(k_1, k_2) = C_{L/2}^{k_2} C_{L-2k_2}^{k_1}$$

Hence, entropy is at its maximum, i.e. $dS = 0$. Although function $\ln(N_{conf})$ is discrete with respect to k_1 and k_2, for large number N_{conf}, this function changes slowly with its arguments. We assume that the number of loci L is sufficiently large to differentiate the function $\ln(N_{conf})$, as if it were continuous

$$0 = dS = \ln\left(\frac{N_{conf}(k_1+1, k_2)}{N_{conf}(k_1, k_2)}\right) dk_1 + \ln\left(\frac{N_{conf}(k_1, k_2+1)}{N_{conf}(k_1, k_2)}\right) dk_2 \qquad (3.5)$$

Function N_{conf} changes with increments in k_1 and k_2 as follows

$$\begin{aligned} N_{conf}(k_1+1, k_2) &\approx \frac{N_{conf}(k_1, k_2)L}{k_1} = \frac{N_{conf}(k_1, k_2)}{f_{01}} \\ N_{conf}(k_1, k_2+1) &\approx \frac{N_{conf}(k_1, k_2)L}{2k_2} = \frac{N_{conf}(k_1, k_2)}{f_{11}} \end{aligned} \qquad (3.6)$$

Here, we neglected small terms in the order of k_i/L by taking into account assumption (3.3). Frequency f_{01} is the haplotype frequency of pairs with the second site mutated, which is equal to f_{10}; and f_{11} is the haplotype frequency of pairs with two sites mutated. In equation (3.6), the haplotype frequencies are calculated in the following way

$$\begin{aligned} f_{01} &= \frac{k_1/2}{L/2} = k_1/L = f_{10} \\ f_{11} &= \frac{k_2}{L/2} = 2k_2/L \end{aligned} \qquad (3.7)$$

Thus, (3.5) takes the form

$$0 = \ln\left(\frac{1}{f_{10}}\right) dk_1 + \ln\left(\frac{1}{f_{11}}\right) dk_2 \qquad (3.8)$$

Return to equation for the fitness (3.4) that ensures us with one more relation for k_1 and k_2. We differentiate equation (3.4) and obtain the additional condition

$$dk_1 = -2(1-E)dk_2 \qquad (3.9)$$

which we substitute in (3.8). Thus, the maximization of entropy is carried out only by one degree of freedom k_2. In result, we obtain the relation between haplotype frequencies and epistatic coefficient E

$$f_{11} = f_{10}^{2(1-E)} \qquad (3.10)$$

Correlation coefficients and the average frequency. Inter-site correlations caused by epistasis are referred to as linkage disequilibrium (LD). Although different definitions of LD that capture different aspects of allelic association have been proposed, here we choose a popular measure of LD proposed by Lewontin [1], which gives 1 if disequilibrium is absent, > 1 if the alleles correlate positively, and < 1 if negatively.

$$D_{11} = \frac{f_{11}}{f^2}, \quad D_{10} = \frac{f_{10}}{f(1-f)} \approx \frac{f_{10}}{f} \tag{3.11}$$

where

$$f = f_{10} + f_{11} \tag{3.12}$$

is the average frequency of mutations per site. Thus, D_{11} is the ratio of the probability of two interacting sites being mutated, to the same probability as if they were independent. Analogously, coefficient D_{10} is the ratio of probabilities for one mutated site. The disadvantage of these expressions is that the average mutant frequency, f, also depends on the epistatic strength. Hence, it would be better to express the correlation coefficients in terms of $k_0/L \equiv f_0$ (Eq. (3.4)), which is a fixed value. Dividing equation (3.4) by L, we obtain f_0

$$f_0 = f_{10} + f_{11}(1-E) \tag{3.13}$$

Equation (3.10) is non-linear in E and cannot be solved analytically. However, due to the presence of a small parameter $f_0 \ll 1$, it can be solved with high asymptotic accuracy in various intervals of E.

At $E = 1/2$, equation (3.10) is simplified to $f_{11} = f_{10}$. Then, equation (3.13) takes the form $f_0 = (3/2)f_{10}$. From equations (3.12) and (3.13), we can express the correlation coefficients and f in terms of f_0

$$D_{11} = \frac{3}{8f_0}, \quad D_{10} = \frac{1}{2}, \quad f = \frac{4}{3}f_0 \quad \left(E = \frac{1}{2}\right) \tag{3.14}$$

Consider the interval

$$0 \le E < \left(\frac{1}{2} - \Delta E\right), \quad \text{where} \quad \Delta E \sim 1/(-2\ln f)$$

Since all the frequencies are small, $f_{11}, f_{10} \ll 1$, we obtain from (3.10) that $f_{11} \ll f_{10}$. Then,

$$f_0 \approx f \approx f_{10}$$

Therefore, from equation (3.10)

$$f_{11} \approx f_0^{2(1-E)}$$

As a result, the approximate correlation coefficients and the average frequency are

$$D_{11} = \frac{1}{f_0^{2E}}, \quad D_{10} = 1, \quad f = f_0 \quad \left(0 < E < \frac{1}{2} - \Delta E\right) \tag{3.15}$$

If there is no epistasis (E=0), $D_{11} = 1$ as expected.

Another interval to consider is $(1/2 + \Delta E) < E < 1$. Now, from (3.10) we have that $f_{10} \ll f_{11}$. Therefore, $f \approx f_{11}$ and $f_0 \approx f_{11}(1 - E)$. Then,

$$f \approx f_{11} \approx f_0/(1 - E)$$

Note that the value of E must not be too close to 1, to keep the value of f small. Otherwise, our consideration does not hold.

From equation (3.10), we obtain

$$f_{10} = \left(\frac{f_0}{1 - E}\right)^{\frac{1}{2(1-E)}}$$

Thus,

$$D_{11} = \frac{1 - E}{f_0}, \quad D_{10} = \left(\frac{f_0}{1 - E}\right)^{\frac{E-1/2}{1-E}}, \quad f = \frac{f_0}{1 - E} \quad \left(\frac{1}{2} + \Delta E' < E < 1\right) \tag{3.16}$$

The plots for D_{11}, D_{10} and f as functions of E (red curves), obtained numerically at $f_0 = 1/100$, are shown on Fig. 7 in the main text.

3.2 Double arches

Here, we re-derive our results for a slightly different configuration that we call "double arches" (see Fig. 6 in the main text): each three loci are connected by two bonds.

Denote the number of double arches with single mutation by k_1, and by k_2 the number of double arches with two *interacting* mutations, k_3 is the number of triplets of mutated sites. The number of double arches with two sites mutated, that do not interact epistatically (no bond between them), must be included in k_1, instead of k_2, as two single mutations.

Configurational entropy is restrained by the fitness given by (1.1), which for the present case can be written in the form

$$k_0 \equiv -\frac{W}{s_0} = k_1 + 2k_2(1 - E) + k_3(3 - 4E) \tag{3.17}$$

Compared to (3.4), equation (3.17) has an additional term, which relates to triplets of mutations, emerging in a given topology. Analogically to the case of isolated pairs, we find the number of combinations in terms of fixed k_1, k_2 and k_3

$$N_{conf} = C^{k_3}_{L/3} C^{k_2}_{L/3-k_3} 2^{k_2} C^{k_1}_{L/3-k_3-k_2} 3^{k_1}$$

To find the maximum of entropy S in k_i, $i = 1, 2, 3$, we calculate the discrete derivative of $\ln(N_{conf})$ in k_i. To do that, we successively increment arguments of N_{conf} and use the assumption that $k_i \ll L$, $i = 1, 2, 3$

$$N_{conf}(k_1 + 1, k_2, k_3) = N_{conf} \frac{3(\frac{L}{3} - k_1 - k_2 - k_3)}{k_1 + 1} \approx N_{conf} \frac{L}{k_1} = \frac{N_{conf}}{f_1} \tag{3.18}$$

Similarly,

$$\begin{aligned} N_{conf}(k_1, k_2 + 1, k_3) &\approx N_{conf} \frac{2L}{3k_2} = \frac{2N_{conf}}{3f_2} \\ N_{conf}(k_1, k_2, k_3 + 1) &\approx N_{conf} \frac{L}{3k_3} = \frac{N_{conf}}{3f_3} \end{aligned} \tag{3.19}$$

where $f_i = k_i/L$.

Then, likewise to the case of isolated pairs, we obtain the relation

$$0 = dS = \ln\left(\frac{1}{f_1}\right) dk_1 + \ln\left(\frac{2}{3f_2}\right) dk_2 + \ln\left(\frac{1}{3f_3}\right) dk_3 \tag{3.20}$$

Differentiating both sides of equation (3.17), we get the condition on dk_1

$$dk_1 = -2(1 - E)dk_2 - (3 - 4E)dk_3 \tag{3.21}$$

We substitute this expression into (3.20) and separate the equation in two

$$\begin{aligned} \frac{2}{3f_2} &= 2(1 - E) \ln \frac{1}{f_1} \\ \frac{1}{3f_3} &= (3 - 4E) \ln \frac{1}{f_1} \end{aligned} \tag{3.22}$$

Thus, we obtain the expressions for f_2 and f_3 in terms of f_1

$$\begin{aligned} f_2 &= \frac{1}{3} f_1^{2(1-E)} \\ f_3 &= \frac{2}{3} f_1^{3-4E} \end{aligned} \tag{3.23}$$

Our goal is to express the correlation coefficients given in (3.11) in terms of E and constant frequency $f_0 = k_0/L$. The latter is obtained by dividing both sides of (3.17) by L

$$f_0 = f_1 + 2(1-E)f_2 + (3-4E)f_3$$

To calculate the correlation coefficients, we need average mutant frequency f, that can be expressed in the form

$$f = f_1 + 2f_2 + 3f_3 \tag{3.24}$$

Frequency of mutated pairs f_{11} should be calculated as follows. There are $k_2 + 2k_3$ epistatic pairs with two mutated sites, divided by the total number of epistatic pairs, $2L/3$. Thus,

$$f_{11} = \frac{3(k_2 + 2k_3)}{2L} = 3\left(\frac{1}{2}f_2 + f_3\right) \tag{3.25}$$

Frequency f_{10} is simply calculated as the difference of f and f_{11}

$$f_{10} = f - f_{11} \tag{3.26}$$

We can write these equations in terms of f_1

$$f_0 = f_1 + \frac{4}{3}(1-E)f_1^{2(1-E)} + \frac{1}{3}(3-4E)f_1^{3-4E}; \tag{3.27}$$

$$f = f_1 + \frac{4}{3}f_1^{2(1-E)} + f_1^{3-4E} \tag{3.28}$$

$$f_{11} = f_1^{2(1-E)} + f_1^{3-4E} \tag{3.29}$$

$$f_{10} = f_1 + \frac{1}{3}f_1^{2(1-E)} \tag{3.30}$$

Now we consider the approximated values of D_{11} and D_{10} for different values of E, provided that all mutation frequencies are much less than zero. At $E = 1/2$, $f_0 = 2f_1$, then $f_1 = f_0/2$. We express all the haplotype frequencies in terms of f_0

$$f_{11} = f_0, \quad f_{10} = 2/3f_0$$

Thus, the correlation coefficients and the average mutant frequency are

$$D_{11} = \frac{9}{25f_0}, \quad D_{10} = \frac{2}{5}, \quad f = \frac{5f_0}{3} \quad \left(E = \frac{1}{2}\right) \tag{3.31}$$

Next, we find the approximate expressions for values D_{11} and D_{10} in interval $[0, 1/2 - \Delta E)$, where $\Delta E \sim 1/(-2\ln f)$. As in this interval

$$f_1 > f_1^{2-2E} > f_1^{3-4E}$$

we can neglect the higher order terms and obtain $f_0 \approx f_1 \approx f_{10} \approx f$, $f_{11} \approx f_1^{2-2E}$. The analytic results for D_{11}, D_{10} and f are

$$D_{11} = \frac{1}{f_0^{2E}}, \quad D_{10} = 1, \quad f = f_0 \quad \left(0 < E < \frac{1}{2} - \Delta E\right) \tag{3.32}$$

At $E = 0$, we obtain $D_{11} \approx 1$ as it was expected in absence of epistasis.

In the interval of E $(1/2 + \Delta E, 3/4)$, we note that the powers of f_1

$$f_1^{3-4E} > f_1^{2-2E} > f_1$$

Then, the expression (3.27) is approximately $f_0 = \frac{1}{3}(3-4E)f_1^{3-4E}$. Thus, we obtain

$$f_1 \approx \left(\frac{3f_0}{3-4E}\right)^{\frac{1}{3-4E}}$$
$$f \approx f_{11} \approx f_1^{3-4E} = \frac{3f_0}{3-4E}$$
$$f_{10} \approx \frac{1}{3}f_1^{2(1-E)} = \frac{1}{3}\left(\frac{3f_0}{3-4E}\right)^{\frac{2(1-E)}{3-4E}}$$

The correlation coefficients and the average mutant frequency have the form

$$D_{11} = \frac{3-4E}{3f_0}, \quad D_{10} = \frac{1}{3}\left(\frac{3-4E}{3f_0}\right)^{\frac{2E-1}{4E-3}}, \quad f = \frac{3f_0}{3-4E} \quad \left(\frac{1}{2} + \Delta E' < E < 1\right) \tag{3.33}$$

The plots for D_{11}, D_{10} and f as functions of E, obtained numerically at $f_0 = 1/100$, are shown on Fig. 7, in the main text (green lines).

3.3 Triple arches

Now we intend to consider the case of isolated double arches with one additional bond, which we call "triple arches" (see Fig. 6b, main text). Thus, all the sites in one cluster interact with each other epistatically.

We keep the notations for k_i and f_i, $i = 1, 2, 3$, introduced in the previous subsection. Equation for the fitness (1.1), in the case of triple arches, takes the form

$$k_0 \equiv -\frac{W}{s_0} = k_1 + 2k_2(1-E) + 3k_3(1-2E) \tag{3.34}$$

As the derivation is similar to the previous case, the details are omitted. The number of combinations of configurations at given k_1, k_2 and k_3 is

$$N_{conf} = C_{L/3}^{k_3} C_{L/3-k_3}^{k_2} 3^{k_2} C_{L/3-k_3-k_2}^{k_1} 3^{k_1}$$

To calculate the discrete derivative of $S = \ln(N_{conf})$ (to maximize entropy by k_i), we increment the arguments of N_{conf}

$$\begin{aligned} N_{conf}(k_1+1, k_2, k_3) &\approx \frac{N_{conf}}{f_1} \\ N_{conf}(k_1, k_2+1, k_3) &\approx \frac{N_{conf}}{f_2} \\ N_{conf}(k_1, k_2, k_3+1) &\approx \frac{N_{conf}}{3f_3} \end{aligned} \tag{3.35}$$

Compared to "'double arches", the only difference is in the second equation: there is no factor of 2/3.

Using (3.35), the derivative of entropy dS can be written as

$$0 = dS = \ln\left(\frac{1}{f_1}\right) dk_1 + \ln\left(\frac{1}{f_2}\right) dk_2 + \ln\left(\frac{1}{3f_3}\right) dk_3 \tag{3.36}$$

On the other hand, the differential of equation (3.34) yields the additional relation

$$dk_1 = -2(1-E)dk_2 - 3(1-2E)dk_3 \tag{3.37}$$

After substitution of (3.37), equation (3.36) can be separated in two

$$\begin{aligned} \ln\frac{1}{f_2} &= 2(1-E)\ln\frac{1}{f_1} \\ \ln\frac{1}{3f_3} &= 3(1-2E)\ln\frac{1}{f_1} \end{aligned} \tag{3.38}$$

Therefore, both frequencies f_2 and f_3 can be expressed in terms of f_1

$$\begin{aligned} f_2 &= f_1^{2(1-E)} \\ f_3 &= \frac{1}{3} f_1^{3(1-2E)} \end{aligned} \tag{3.39}$$

Correlation coefficients and the average mutant frequency. The number of epistatic pairs is equal to L. The haplotype frequencies can be expressed as follows

$$\begin{aligned} f_{11} &= \frac{k_2 + 3k_3}{L} = f_2 + 3f_3 \\ f_{10} = f_{01} &= \frac{k_1 + k_2}{L} = f_1 + f_2 \end{aligned} \tag{3.40}$$

The average mutant frequency and constant frequency $f_0 = k_0/L$ are given by

$$\begin{aligned} f &= f_{11} + f_{10} = f_1 + 2f_2 + 3f_3 \\ f_0 &= f_1 + 2(1-E)f_2 + 3(1-2E)f_3 \end{aligned} \tag{3.41}$$

Next, we can write all the expressions in terms of f_1

$$\begin{aligned} f_0 &= f_1 + 2(1-E)f_1^{2(1-E)} + (1-2E)f_1^{3(1-2E)} \\ f &= f_1 + 2f_1^{2(1-E)} + f_1^{3(1-2E)} \\ f_{11} &= f_1^{2(1-E)} + f_1^{3(1-2E)} \\ f_{10} &= f_1 + f_1^{2(1-E)} \end{aligned} \tag{3.42}$$

We find approximate values for D_{11} and D_{10} for different values of E in interval $[0, 1/2)$. We can not take point $E = 1/2$, since from equation (3.39), we have $f_3 = 1/3$, which does not satisfy condition $f_i \ll 1$.

Consider the equations at $E = 1/4$. The powers $(3-6E)$ and $(2-2E)$ become equal to each other, that yields $f_{11} = 2f_1^{3/2}$. Using the condition $f_1 \ll 1$, we obtain the relation

$$f_0 = f \approx f_1 \approx f_{10} \tag{3.43}$$

That results in

$$D_{11} \approx \frac{2}{\sqrt{f_0}}, \quad D_{10} \approx 1, \quad f = f_0 \quad (E = 1/4) \tag{3.44}$$

At $E = 1/3$, we note that the power $3(1-2E)$ is equal to 1. Then, we obtain the approximation

$$f_{11} \approx f_{10} \approx f_1$$

which gives $f \approx 2f_1$, $f_0 \approx 4/3f_1$ and $f_1 = 3/4f_0$. It yields to

$$f = \frac{3}{2}f_0, \quad D_{11} \approx \frac{1}{3f_0}, \quad D_{10} \approx \frac{1}{2} \quad (E = 1/3) \tag{3.45}$$

Let us now consider the interval of E $(0, 1/4 - \Delta E)$, where $\Delta E \sim 1/(-2\ln f)$. We obtain relations (3.43) and $f_1 \approx f_1^{2(1-E)}$. Thus,

$$f = f_0, \quad D_{11} \approx \frac{1}{f_0^{2E}}, \quad D_{10} \approx 1 \tag{3.46}$$

As expected, at $E = 0$, $D_{11} \approx 1$.

In interval $(1/4+\Delta E, 1/3-\Delta E')$, where $\Delta E' \sim 1/(-6\ln f)$, we have

$$f_1^{2-2E} \ll f_1^{3-6E} \ll f_1$$

and then relation (3.43) holds again. The frequency of double mutations can be approximated as $f_{11} \approx f_1^{3-6E}$. It results in

$$f = f_0, \quad D_{11} \approx \frac{1}{f_0^{6E-1}}, \quad D_{10} \approx 1 \tag{3.47}$$

Finally, in interval $(1/3+\Delta E', 1/2)$, we can see that

$$f_1^{2-2E} \ll f_1 \ll f_1^{3-6E}$$

Using this notice, we obtain relations $f_{10} \approx f_1$, $f \approx f_{11}$ and $f_0 = (1-2E)f$. Thus, the correlation coefficients and the average mutant frequency have the form

$$D_{11} = \frac{1-2E}{f_0}, \quad D_{10} = \left(\frac{1-2E}{f_0}\right)^{\frac{2(1-3E)}{3(1-2E)}}, \quad f = \frac{f_0}{1-2E} \tag{3.48}$$

The plots for D_{11}, D_{10} and f as functions of E, obtained numerically at $f_0 = 1/100$, can be found in Fig. 7 in the main text (black curves).

3.4 Connected long chain

In this subsection, we consider a long cluster, whose sites are linked by epistasis in a chain (shown in Fig. 6d, main text). Since mutations are assumed to be rare, in such a long chain, we would observe sub-chains that can be grouped by number of mutated sites i, where $i = 1, \ldots, I$, and $1 \ll I \ll L$. We assume that the system of sub-clusters is sparse, so we need to introduce I, the upper limit of the cluster size. Further, we will see that the result does not depend on this parameter. The number of clusters in a group i is denoted by k_i.

Configurational entropy is under restriction of fitness (1.1), which for the case of a long chain takes the form

$$k_0 = k_1 + \sum_{i=2}^{I}(i - 2(i-1)E)k_i \tag{3.49}$$

The approximate number of combinations of possible sequences at given k_i can be written as

$$N_{conf} = \prod_{i=1}^{I} C_L^{k_i} \tag{3.50}$$

provided that $\sum_{i=1}^{I} k_i \ll L$. In (3.50), we neglected overlaps of sub-clusters. To maximize entropy by k_i, we find the derivative of $\ln(N_{conf})$ similarly to the previous cases. We write it directly and equate it to zero to find the maximum of S

$$0 = dS = \ln\left(\frac{1}{f_1}\right) dk_1 + \sum_{k=2}^{I} \ln\left(\frac{1}{f_i}\right) dk_i \tag{3.51}$$

where $f_i = k_i/L$.

The differential of (3.49) yields the condition on dk_1

$$dk_1 = -\sum_{i=2}^{I} (i - 2(i-1)E) dk_i \tag{3.52}$$

After substitution of this expression into (3.51), we obtain

$$-\ln\left(\frac{1}{f_1}\right) \sum_{i=2}^{I} (i - 2(i-1)E) dk_i + \sum_{k=2}^{I} \ln\left(\frac{1}{f_i}\right) dk_i = 0 \tag{3.53}$$

As a result, the expressions for frequencies f_i in terms of f_1 are

$$f_i = f_1^{i-2(i-1)E} = f_1^{2E} \left(f_1^{1-2E}\right)^i \tag{3.54}$$

Correlation coefficients and the average frequency. Constant frequency f_0 can be obtained dividing (3.49) by L

$$f_0 = \sum_{i=1}^{I} (i - 2(i-1)E) f_i \tag{3.55}$$

The average frequency and the frequency of double mutations can be calculated as follows

$$\begin{aligned} f &= f_1 + 2f_2 + \ldots = \sum_{i=1}^{I} i f_i \\ f_{11} &= f_2 + 2f_3 + \ldots = \sum_{i=2}^{I} (i-1) f_i \end{aligned} \tag{3.56}$$

Then, as $f = f_{10} + f_{11}$, frequency f_{10} is the difference of frequencies f and f_{11}. That yields

$$f_{10} = \sum_{i=1}^{I} f_i \tag{3.57}$$

Using (3.54), we express f_i in terms of f_1 for all the relations

$$f_0 = \sum_{i=1}^{I} f_1^{i-2(i-1)E}(i - 2(i-1)E) \tag{3.58}$$

$$f = f_1^{2E} \sum_{i=1}^{I} i f_1^{i(1-2E)} \tag{3.59}$$

$$f_{11} = f_1^{2E} \sum_{i=1}^{I} (i-1) f_1^{i(1-2E)} \tag{3.60}$$

$$f_{10} = f_1^{2E} \sum_{i=1}^{I} f_1^{i(1-2E)} \tag{3.61}$$

Frequency f_{10} can be calculated using the formula for geometric progression with common ratio f_1^{1-2E}

$$f_{10} = f_1^{2E} \left(f_1^{1-2E} \frac{1 - f_1^{(1-2E)I}}{1 - f_1^{1-2E}} \right) \approx \frac{f_1}{1 - f_1^{1-2E}} \tag{3.62}$$

provided that $E < 1/2$. In the expressions for f_0 and f, there are sums that can transformed as follows

$$\sum_{i=1}^{I} i a_i = \sum_{i=1}^{I} i e^{i \ln a} = \frac{d}{d(\ln a)} \sum_{i=1}^{I} e^{i \ln a} = \frac{d}{d(\ln a)} \sum_{i=1}^{I} a_i$$

Using this remark, we obtain

$$f_0 = \frac{d}{d(\ln f_1)} \left(\frac{f_1}{1 - f_1^{1-2E}} \right) \tag{3.63}$$

$$f = \frac{f_1^{2E}}{(1-2E)} \frac{d}{d(\ln f_1)} \left(\frac{f_1^{1-2E}}{1 - f_1^{1-2E}} \right) \tag{3.64}$$

For convenience, we introduce the notation

$$x = (1-2E) \ln(1/f_1) \tag{3.65}$$

Then, $e^{-x} = f_1^{1-2E}$ and $d/d(\ln f_1) = -(1-2E)d/dx$. We can rewrite all the

frequencies in terms of x

$$f_0 = \frac{e^{-\frac{x}{1-2E}}(1-2Ee^{-x})}{(1-e^{-x})^2} \tag{3.66}$$

$$f = \frac{e^{-\frac{x}{1-2E}}}{(1-e^{-x})^2} \tag{3.67}$$

$$f_{10} = \frac{e^{-\frac{x}{1-2E}}}{1-e^{-x}} \tag{3.68}$$

$$f_{11} = f - f_{10} = \frac{e^{-\frac{2(x-E)}{1-2E}}}{(1-e^{-x})^2} \tag{3.69}$$

First, we consider the case of $x \gg 1$, which is equivalent to the case of small values of epistatic coefficient

$$E \ll \frac{1 - \frac{1}{ln(1/f_1)}}{2}$$

Then, we can neglect small terms and obtain the relations

$$f_0 \approx f \approx f_{10} \approx f_1, \quad f_{11} = f_0^{2-2E}$$

Thus, the correlation coeffcients and the average mutant frequency are in the form

$$D_{11} \approx \frac{1}{f_0^{2E}}, \quad D_{10} \approx 1, \quad f \approx f_0 \tag{3.70}$$

Now let us consider $x \ll 1$, that corresponds to the case of E close to 1/2. Then $(1-2E) \ll 2Ex$, and frequency f_0 can be approximated as follows

$$f_0 \approx \frac{2Exf_1}{(1-e^{-x})^2} \approx \frac{xf_1}{(1-e^{-x})^2} \approx \frac{xf_1}{x^2} \approx \frac{f_1}{x} \tag{3.71}$$

By expressing frequency f_1 in terms of f_0 and substituting x by expression (3.65), we obtain the implicit equation for f_1

$$f_1 = f_0(1-2E)\ln\left(\frac{1}{f_1}\right) \tag{3.72}$$

whose approximate solution is

$$f_1 = f_0(1-2E)\ln\left(\frac{1}{(1-2E)f_0}\right) \tag{3.73}$$

Then,

$$x = (1-2E)\ln\left(\frac{1}{(1-2E)f_0}\right) \tag{3.74}$$

Approximated results for f, f_{10} and f_{11}

$$f \approx \frac{f_1}{x^2} \approx \frac{f_0}{(1-2E)\ln\left(\frac{1}{(1-2E)f_0}\right)}$$
$$f_{10} = \frac{f_1}{1-e^{-x}} \approx \frac{f_1}{x} \approx f_0 \tag{3.75}$$
$$f_{11} = \frac{f_1^{2(1-E)}}{(1-e^{-x})^2} \approx \frac{f_1}{x^2} \approx f$$

Consequently, the correlation coefficients and the average frequency are as follows

$$D_{11} = \frac{(1-2E)\ln\left(\frac{1}{(1-2E)f_0}\right)}{f_0}, \quad D_{10} = (1-2E)\ln\left(\frac{1}{(1-2E)f_0}\right) \tag{3.76}$$
$$f = \frac{f_0}{(1-2E)\ln\left(\frac{1}{(1-2E)f_0}\right)} \tag{3.77}$$

Refer to Fig. 7 in the main text, to see the corresponding plots for D_{11}, D_{10} and f as functions of E (shown in magenta), obtained numerically at $f_0 = 1/100$.

3.5 Large binary tree

Now we are interested in more complicated configuration, when all sites of the genome are linked by epistasis in one cluster, such that each site interacts with three other loci. We consider the special case when the network is equivalent to a binary tree, i.e. any two sites are connected only by one path.

Remark. Note that for that topology, equation for fitness (1.1) and the expressions for frequencies f, f_{11} and f_{10} (in terms of f_i) have the same forms as in corresponding equations (3.49), (3.56) and (3.57), given for a long chain. This is due to the fact that for a binary tree, as well as for a chain, for every i sites there are $i-1$ bonds.

Similar to the previous case, we can group sub-trees by number of mutated nodes i. Denote by k_i the number of i-trees. The number of possible configurations can be estimated as follows

$$N_{conf} = C_L^{k_1} C_{L_2}^{k_2} (A_2)^{k_2} \dots C_{L_i}^{k_i} (A_i)^{k_i} \dots = \prod_{i=1}^{I} C_{L_i}^{k_i} (A_i)^{k_i}$$

As mutations are assumed to be rare, we neglected the overlaps of sub-trees. Each term $C_{L_i}^{k_i}$ is the number of combinations to put k_i roots on L_i vertices, where L_i, in turn, depends on the height of the i-tree. We multiply this number of combinations by factor $(A_i)^{k_i}$, since there are A_i possible forms for each i-tree, where

$$A_i = \frac{(2i)!}{i!(i+1)!}, \quad A_i \sim \frac{4^i}{i^{3/2}\sqrt{\pi}} \quad \text{for } i \gg 1 \tag{3.78}$$

For convenience, we introduce some notations

$$\beta_i = \frac{L_i}{L}, \quad A_i = 4^i \alpha_i, \quad \text{where} \quad \alpha_i = \frac{(2i)!}{4^i i!(i+1)!} \tag{3.79}$$

The condition at the maximum of entropy have the form

$$0 = dS = \sum_{i=1}^{I} \ln\left(\frac{N_{conf}(\ldots, k_i+1, \ldots)}{N_{conf}}\right) dk_i = \sum_{i=1}^{I} \ln\left(\frac{A_i \beta_i}{f_i}\right) dk_i \tag{3.80}$$

where $f_i = k_i/L$. After substitution of dk_1 from equation (3.52), which hold for the present case as well, we obtain

$$\sum_{i=2}^{I} \ln\left(\frac{A_i \beta_i}{f_i}\right) dk_i - \ln\left(\frac{1}{f_1}\right) \sum_{i=2}^{I} (i - 2(i-1)E) dk_i = 0 \tag{3.81}$$

As $L_1 = L$, frequencies f_i can be expressed in terms of f_1 in the form

$$f_i = A_i \beta_i f_1^{i-2(i-1)E} = \alpha_i \beta_i f_1^{2E} \left(4 f_1^{1-2E}\right)^i \tag{3.82}$$

Frequencies f_i must be less than 1. Consider the asymptotic behavior of expression (3.82). For large i, coefficient $\alpha_i \sim 1/i^{3/2}$, and coefficient $\beta_i \sim 2L/(i+1)$, if we consider a perfect (the more branched) binary tree. The second asymptotic was obtained from the estimation of height h of a perfect binary tree in terms of number of nodes i

$$h = log_2(i+1) - 1$$

Then, the estimation of the number of sites of i-tree in terms of the height is $L_i = 2^h$. Thus, we obtain the estimation of coefficients $\beta_i = L_i/L$. As in our case, the tree does not have to be perfect, we estimate β_i as for a binary tree whose nodes can have either one or two children:

$$\beta_i = \frac{2L}{(i+1)^\alpha}, \quad \text{where} \quad \alpha \in (1, 2)$$

Thus, we can see that expression (3.82), for $i \gg 1$, is mainly governed by coefficient $4f_1^{1-2E}$. Therefore, we need to provide $4f_1^{1-2E} < 1$ which is equivalent to

$$E < E', \quad \text{where} \quad E' = \frac{1}{2}\left(1 - \frac{\ln 4}{\ln \frac{1}{f_1}}\right)$$

However, we obtained numerically (see Fig. 7 of the main text) that the curves of correlation coefficients and mutant frequency could be continued until point $E_c = 1/2$. This is due to the contribution of coefficients α_i and β_i, that appear to still be important in interval $(E', 1/2)$, slowing down the divergence of expression (3.82).

Correlation coefficients and the average mutant frequency. As we noted earlier, frequencies f_0, f, f_{11} and f_{10} have the same form as in corresponding equations (3.55), (3.56) and (3.57), obtained for the case of a long chain. Rewrite these expression in terms of f_1, using (3.82), and introduce notation $e^{-x} = 4f_1^{1-2E}$

$$f_0 = f_1^{2E} \sum_{i=1}^{I} \alpha_i \beta_i e^{-ix}(i - 2(i-1)E) \tag{3.83}$$

$$f = f_1^{2E} \sum_{i=1}^{I} i\alpha_i \beta_i e^{-ix} \tag{3.84}$$

$$f_{11} = f_1^{2E} \sum_{i=1}^{I} (i-1)\alpha_i \beta_i e^{-ix} \tag{3.85}$$

$$f_{10} = f_1^{2E} \sum_{i=1}^{I} \alpha_i \beta_i e^{-ix} \tag{3.86}$$

Note, that for $i \gg 1$,

$$\alpha_i \beta_i e^{-ix} = \frac{e^{-ix}}{i^{3/2+\alpha}} \tag{3.87}$$

Consider the case when $x \gg 1$, which is equivalent to $E \ll E_c$. Since the right-hand side of (3.87) converges very fast with large x, we take only the first non-zero elements of sums (3.83), (3.84), (3.85), (3.86). Thus, we obtain that

$$f_0 \approx f \approx f_{10} \approx f_1, \quad f_{11} = f_0^{2-2E}$$

Then,

$$D_{11} \approx \frac{1}{f_0^{2E}}, \quad D_{10} \approx 1, \quad f \approx f_0 \tag{3.88}$$

In case $x \ll 1$ (equivalent to $E \sim E'$), the sum

$$\sum_{i=1}^{I} \frac{e^{-ix}}{i^{3/2+\alpha}} \approx \sum_{i=1}^{I} \frac{1}{i^{3/2+\alpha}} \tag{3.89}$$

converges for $i > 1$, if $I \to \infty$. We can approximate this sum by the sum of finite number of elements. We take 50 elements, where β_i are taken as follows

$$\beta_1 = 1, \quad \beta_2 = \beta_3 = 1/2, \quad \beta_i = \frac{2}{2^{log_{1.5}(i+1)}} \quad i > 3 \tag{3.90}$$

and obtain

$$f_0 = \sum_{i=1}^{50} (i - 2(i-1)E) f_i \approx \sum_{i=1}^{50} f_i = f_{10} \approx 1.48 f_1 \tag{3.91}$$

$$f = \sum_{i=1}^{50} i f_i \approx 2.49 f_1 \tag{3.92}$$

$$f_{11} = \sum_{i=1}^{50} (i-1) f_i \approx 1.01 f_1 \tag{3.93}$$

and frequency $f_{10} = f - f_{11} = 1.48 f_1$. Then, the approximate correlation coefficients and the average mutant frequency in terms of f_0 are

$$D_{11} \approx \frac{1}{4 f_0}, \quad D_{10} \approx 0.6, \quad f \approx 1.68 f_0 \tag{3.94}$$

The plots for D_{11}, D_{10} and f as functions of E (blue curves), obtained numerically at $f_0 = 1/100$, approximated by the sums of 50 elements f_i are shown in Fig. 7 of the main text.

3.6 Double arches with unequal interactions

So far, we investigated the network topologies, assuming that all epistatic interactions are equal. As real systems are more complex, it is also important to study how the result would change if several levels of epistatic strength were present. Here we touch on this issue by considering a simple case of network topology.

Consider again the topology of "double arches" (Fig. 6c in the main text), but now assuming that left bonds have epistatic strength E and right ones have $E/2$. We keep notations k_1 and k_3 for numbers of singles and triplets.

However, in the present case, we need to introduce two different notations k_2 and k'_2 for numbers of doubles interacting with epistatic strengths E and $E/2$, respectively. Then, fitness equation (1.1) can be written in terms of k_1, k_2, k'_2 and k_3 in the form

$$k_0 = k_1 + 2k_2(1-E) + k'_2(2-E) + 3k_3(1-E) \tag{3.95}$$

Next, we calculate the number of possible combinations in terms of k_1, k_2, k'_2 and k_3

$$N_{conf} = C_{L/3}^{k_3} C_{L/3-k_3}^{k_2} C_{L/3-k_3-k_2}^{k'_2} C_{L/3-k_3-k_2-k'_2}^{k_1} 3^{k_1}$$

Analogically to the previous cases, we increment the arguments of $N_{conf}(k_1, k_2, k'_2, k_3)$ and approximate the resulted values, taking into account that $k_i \ll L$

$$\begin{aligned} N_{conf}(k_1+1, k_2, k'_2, k_3) &\approx \frac{N_{conf}}{f_1} \\ N_{conf}(k_1, k_2+1, k'_2, k_3) &\approx \frac{N_{conf}}{3f_2} \\ N_{conf}(k_1, k_2, k'_2+1, k_3) &\approx \frac{N_{conf}}{3f'_2} \\ N_{conf}(k_1, k_2, k'_2, k_3+1) &\approx \frac{N_{conf}}{3f_3} \end{aligned} \tag{3.96}$$

Here we used the notations for f_1, f_2 and f_3 given previously, and introduce $f'_2 \equiv k'_2/L$.

Analogously to the previous cases, we assume a stationary state. Then, the maximum of entropy yields $dS = 0$. Using discrete derivative of $\ln(N_{conf})$, we obtain the following relation

$$0 = dS = \ln\left(\frac{1}{f_1}\right) dk_1 + \ln\left(\frac{1}{3f_2}\right) dk_2 + \ln\left(\frac{1}{3f'_2}\right) dk'_2 + \ln\left(\frac{1}{3f_3}\right) dk_3 \tag{3.97}$$

From equation (3.95), we have

$$dk_1 = -2(1-E)dk_2 - (2-E)dk'_2 - 3(1-E)dk_3 \tag{3.98}$$

Now, we can substitute dk_1 in (3.97) by the relation (3.98). Thus, equation (3.97) can be separated in three

$$\begin{aligned} \ln\frac{1}{3f_2} &= 2(1-E)\ln\frac{1}{f_1} \\ \ln\frac{1}{3f'_2} &= (2-E)\ln\frac{1}{f_1} \\ \ln\frac{1}{3f_3} &= 3(1-E)\ln\frac{1}{f_1} \end{aligned} \tag{3.99}$$

Then, frequencies f_2, f_2' and f_3 can be expressed in terms of f_1

$$
\begin{aligned}
f_2 &= \frac{1}{3} f_1^{2(1-E)} \\
f_2' &= \frac{1}{3} f_1^{(2-E)} \\
f_3 &= \frac{1}{3} f_1^{3(1-E)}
\end{aligned}
\tag{3.100}
$$

Correlation coefficients and the average mutant frequency. The mutant frequency has the form

$$
f = \frac{k_1 + 2k_2 + 2k_2' + 3k_3}{L} = f_1 + 2f_2 + 2f_2' + 3f_3 \tag{3.101}
$$

As the number of epistatic interactions is equal to $2L/3$, the haplotype frequencies can be found as follows

$$
\begin{aligned}
f_{11} &= \frac{3(k_2 + k_2' + 2k_3)}{2L} = \frac{3}{2}(f_2 + f_2') + 3f_3 \\
f_{10} &= f - f_{11} = f_1 + \frac{1}{2}\left(f_2 + f_2'\right)
\end{aligned}
\tag{3.102}
$$

Frequency related to fitness $f_0 = k_0/L$ is found from (3.95)

$$
f_0 = f_1 + 2(1-E)f_2 + (2-E)f_2' + 3(1-E)f_3 \tag{3.103}
$$

Next, we can write all the expressions in terms of f_1

$$
\begin{aligned}
f_{11} &= \frac{1}{2}\left(f_1^{2(1-E)} + f_1^{2-E}\right) + f_1^{3(1-E)} \\
f &= f_1 + \frac{2}{3}\left(f_1^{2(1-E)} + f_1^{2-E}\right) + f_1^{3(1-E)} \\
f_{10} &= f_1 + \frac{1}{6}\left(f_1^{2(1-E)} + f_1^{2-E}\right) \\
f_0 &= f_1 + \frac{2}{3}(1-E)f_1^{2(1-E)} + \frac{2-E}{3} f_1^{2-E} + (1-E) f_1^{3(1-E)}
\end{aligned}
\tag{3.104}
$$

Consider equations (3.104) at $E = 1/2$. Note that at this point

$$
f_1 = f_1^{2(1-E)}, \quad f_1^{2-E} = f_1^{3(1-E)} = f_1^{3/2}, \quad f_1^{2(1-E)} \gg f_1^{2-E}
$$

These relations show that clusters with single and doubles mutated sites with epistatic strength E, outnumber triplets and doubles with epistatic strength

$E/2$. Thus, equations (3.104) can be approximated, as follows

$$\begin{aligned} f &\approx f_1 + \frac{2}{3}f_1 = \frac{5}{3}f_1 \\ f_{11} &\approx \frac{1}{2}f_1 \\ f_{10} &\approx f_1 + \frac{1}{6}f_1 = \frac{7}{6}f_1 \\ f_0 &\approx f_1 + \frac{1}{3}f_1 = \frac{4}{3}f_1 \end{aligned} \tag{3.105}$$

Frequency $f_1 = (3/5)f$. Now, we can express all the frequencies in terms of mutant frequency f

$$\begin{aligned} f_{11} &\approx \frac{3}{10}f \\ f_{10} &\approx f_1 + \frac{1}{6}f_1 = \frac{7}{10}f \\ f_0 &\approx f_1 + 1/3f_1 = \frac{4}{5}f \end{aligned} \tag{3.106}$$

Thus, correlation coefficients and mutant frequency (in terms of f_0) are found

$$D_{11} = \frac{3}{10f}, \quad D_{10} \approx \frac{7}{10}, \quad f = \frac{5}{4}f_0, \quad E = \frac{1}{2} \tag{3.107}$$

Consider equations (3.104) at another important point $E = 2/3$. We have

$$f_1^{2(1-E)} = f_1^{2/3}, \quad f_1^{2-E} = f_1^{4/3}, \quad f_1 = f_1^{3(1-E)}, \quad f_1^{2(1-E)} \gg f_1$$

It shows that doubles with epistatic strength E outnumber singles and triplets, which have frequencies of the same order. The most rare are doubles with epistatic strength $E/2$. Thus, we approximate the frequencies, as follows

$$\begin{aligned} f &\approx \frac{2}{3}f_1^{2/3}, \quad f_0 = \frac{2}{3}(1-E)f_1^{2/3} = \frac{1}{3}f \\ f_{10} &\approx \frac{1}{6}f_1^{2/3} = \frac{1}{4}f, \quad f_{11} \approx \frac{1}{2}f_1^{2/3} = \frac{3}{4}f \end{aligned} \tag{3.108}$$

The correlation coefficients and the mutant frequency have the form

$$D_{11} = \frac{3}{4f}, \quad D_{10} = \frac{1}{4}, \quad f = 3f_0, \quad E = \frac{2}{3} \tag{3.109}$$

Consider now equations (3.104) in the interval of $E \in [0, 1/2 - \Delta E_1)$. In this interval

$$f_1 \gg f_1^{2(1-E)} \gg f_1^{2-E} \gg f_1^{3(1-E)}$$

we approximate the frequencies given by (3.104), as follows

$$\begin{aligned} f &\approx f_1 \approx f_{10} \approx f_0 \\ f_{11} &\approx \frac{1}{2} f_1^{2(1-E)} \end{aligned} \tag{3.110}$$

Then, we obtain the correlation coefficients and the mutant frequency in the form

$$D_{11} \approx \frac{1}{2f^{2E}}, \quad D_{10} \approx 1, \quad f = f_0, \quad E \in \left[0, \frac{1}{2} - \Delta E_1\right) \tag{3.111}$$

In interval $(1/2 + \Delta E_2, 2/3 - \Delta E_3)$, we note that

$$f_1^{2-2E} \gg f_1 \gg f_1^{3(1-E)} \gg f_1^{2-E}$$

Thus, we approximate the relations

$$\begin{aligned} f &\approx \frac{2}{3} f_1^{2(1-E)}, \quad f_0 = \frac{2}{3}(1-E) f_1^{2(1-E)} \approx (1-E) f \\ f_{10} &\approx \frac{1}{6} f_1^{2(1-E)} \approx \frac{1}{4} f, \quad f_{11} \approx \frac{1}{2} f_1^{2(1-E)} \approx \frac{3}{4} f \end{aligned} \tag{3.112}$$

and the correlation coefficients and the average mutant frequency have the form

$$D_{11} = \frac{3}{4f}, \quad D_{10} = \frac{1}{4}, \quad f = \frac{f_0}{1-E}, \quad E \in \left(\frac{1}{2} + \Delta E_2, \frac{2}{3} - \Delta E_3\right) \tag{3.113}$$

Finally, consider the interval of E $(2/3 + \Delta E_4, 1)$. Terms in equations (3.104) are ordered, as follows

$$f_1^{3(1-E)} \gg f_1^{2(1-E)} \gg f_1 \gg f_1^{2-E}$$

Thus, we approximate

$$\begin{aligned} f &\approx f_1^{3(1-E)}, \quad f_0 = (1-E) f_1^{3(1-E)} \approx (1-E) f \\ f_{10} &\approx \frac{1}{6} f_1^{2(1-E)} = \frac{1}{6} f^{2/3}, \quad f_{11} \approx f_1^{3(1-E)} \approx f \end{aligned} \tag{3.114}$$

The correlation coefficients and the mutant frequency have the form

$$D_{11} \approx \frac{1}{f}, \quad D_{10} \approx \frac{1}{6f^{1/3}}, \quad f = \frac{f_0}{1-E}, \quad E \in \left(\frac{2}{3} + \Delta E_4, 1\right) \tag{3.115}$$

To compare results for double arches with equal and unequal interactions, the plots for $D_{11}(E)$, $D_{10}(E)$, $f(E)$ and UFE relation obtained numerically are shown in Fig S3.

4 Supplementary references

[1] Lewontin, R. C. (1964). The interaction of selection and linkage. I. General considerations; heterotic models. Genetics. 49 (1): 49-67.
