# Supplementary material for: Evolutionary footprint of epistasis
Source: PLoS Comput Biol. 2018 Sep 17;14(9):e1006426. doi: 10.1371/journal.pcbi.1006426 (PMC6177197; doi:10.1371/journal.pcbi.1006426)
Supplement: S1 Table — Note that in the expression for exponential of entropy we neglect ki compared to L. (PDF) [file pcbi.1006426.s002.pdf]

Table 2: **Correlation coefficients and critical points for five examples of network topology** (see Fig. 6, main text). b_i is the number of bonds for a cluster of i mutations, E_c is the point of full compensation, E_{UFE} is the maximum epistatic strength at which UFE still applies. D_{11} and D_{10} are pairwise correlation coefficients, f is mutant allelic frequency.

Name	Fig	Bond #	E_c	E_{UFE}	Interval E	D_{11}	D_{10}	$f(E)$
Arch	6a	b_2=1	1	1	$E < \frac{1}{2}$<br>$\frac{1}{2} < E < 1$	f^{-2E}<br>f^{-1}	1<br>$f^{\frac{E-\frac{1}{2}}{1-E}}$	f_0<br>$\frac{f_0}{1-E}$
Triple arch	6b	b_3=3	1/2	1/4	$E < \frac{1}{4}$<br>$\frac{1}{4} < E < \frac{1}{3}$<br>$\frac{1}{3} < E < \frac{1}{2}$	f^{-2E}<br>f^{1-6E}<br>f^{-1}	1<br>1<br>$f^{\frac{E-1/3}{1/2-E}}$	f_0<br>f_0<br>$\frac{f_0}{1-2E}$
Double arch	6c	b_3=2	3/4	1/2	$E < \frac{1}{2}$<br>$\frac{1}{2} < E < \frac{3}{4}$	f^{-2E}<br>f^{-1}	1<br>$\frac{1}{3} f^{\frac{2E-1}{3-4E}}$	f_0<br>$\frac{f_0}{1-4E/3}$
Chain	6d	$b_i = i$-1	1/2	$\frac{1}{2} - \frac{1}{\log\frac{1}{f_0}}$	$\frac{1}{2} - E \gg \frac{1}{\log\frac{1}{f_0}}$<br>$\frac{1}{2} - E \ll \frac{1}{\log\frac{1}{f_0}}$	f^{-2E}<br>f^{-1}	1<br>$\frac{f_0}{f}$	f_0<br>$\frac{f_0}{(1-2E)\log\frac{1}{f_0(1-2E)}}$
Binary tree	6e	$b_i = i$-1	1/2	$\frac{1}{2} - \frac{\log 4}{\log\frac{1}{f_0}}$	$E_{UFE} - E \gg \frac{1}{\log\frac{1}{f_0}}$<br>$E_{UFE} - E \ll \frac{1}{\log\frac{1}{f_0}}$	f^{-2E}<br>f^{-1}	1<br>–	f_0<br>–
Double arch, uneq.	6c	b_3=2	1	1	$E < \frac{1}{2}$<br>$\frac{1}{2} < E < \frac{2}{3}$<br>$\frac{2}{3} < E < 1$	$\frac{1}{2} f^{-2E}$<br>$\frac{3}{4} f^{-1}$<br>f^{-1}	1<br>$\frac{1}{4}$<br>$\frac{1}{6} f^{-\frac{1}{3}}$	f_0<br>$\frac{f_0}{1-E}$<br>$\frac{f_0}{1-E}$
