# Supplementary figures and images for: Evolutionary footprint of epistasis
Source: PLoS Comput Biol. 2018 Sep 17;14(9):e1006426. doi: 10.1371/journal.pcbi.1006426 (PMC6177197; doi:10.1371/journal.pcbi.1006426)

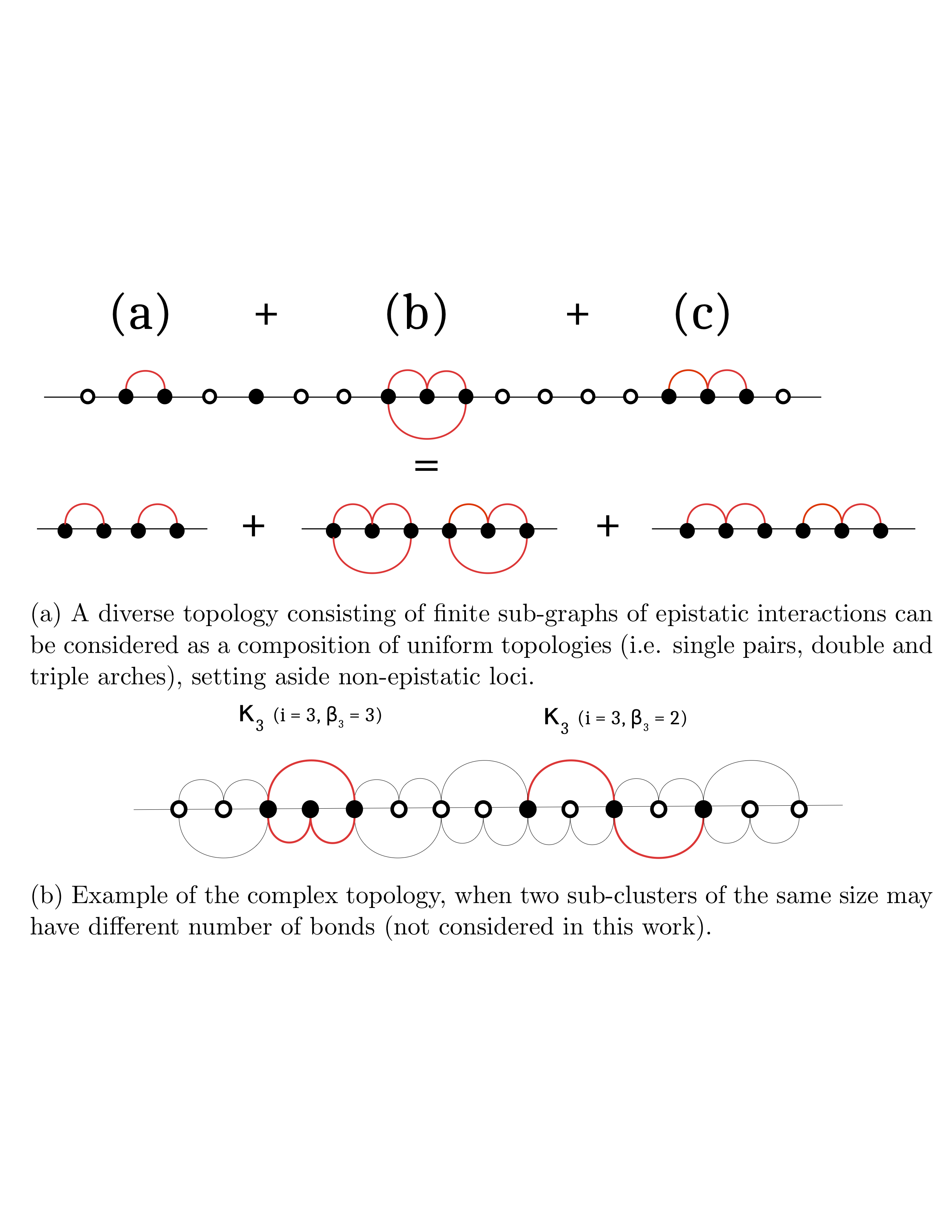

Supplement: S1 Fig — (a) A diverse topology consisting of finite sub-graphs of epistatic interactions can be considered as a composition of uniform topologies (i.e. single pairs, double and triple arches), setting aside non-epistatic loci. (b) Example of the complex topology, when two sub-clusters of the same size may have different number of bonds (not considered in this work). (TIFF) [file pcbi.1006426.s004.tiff]

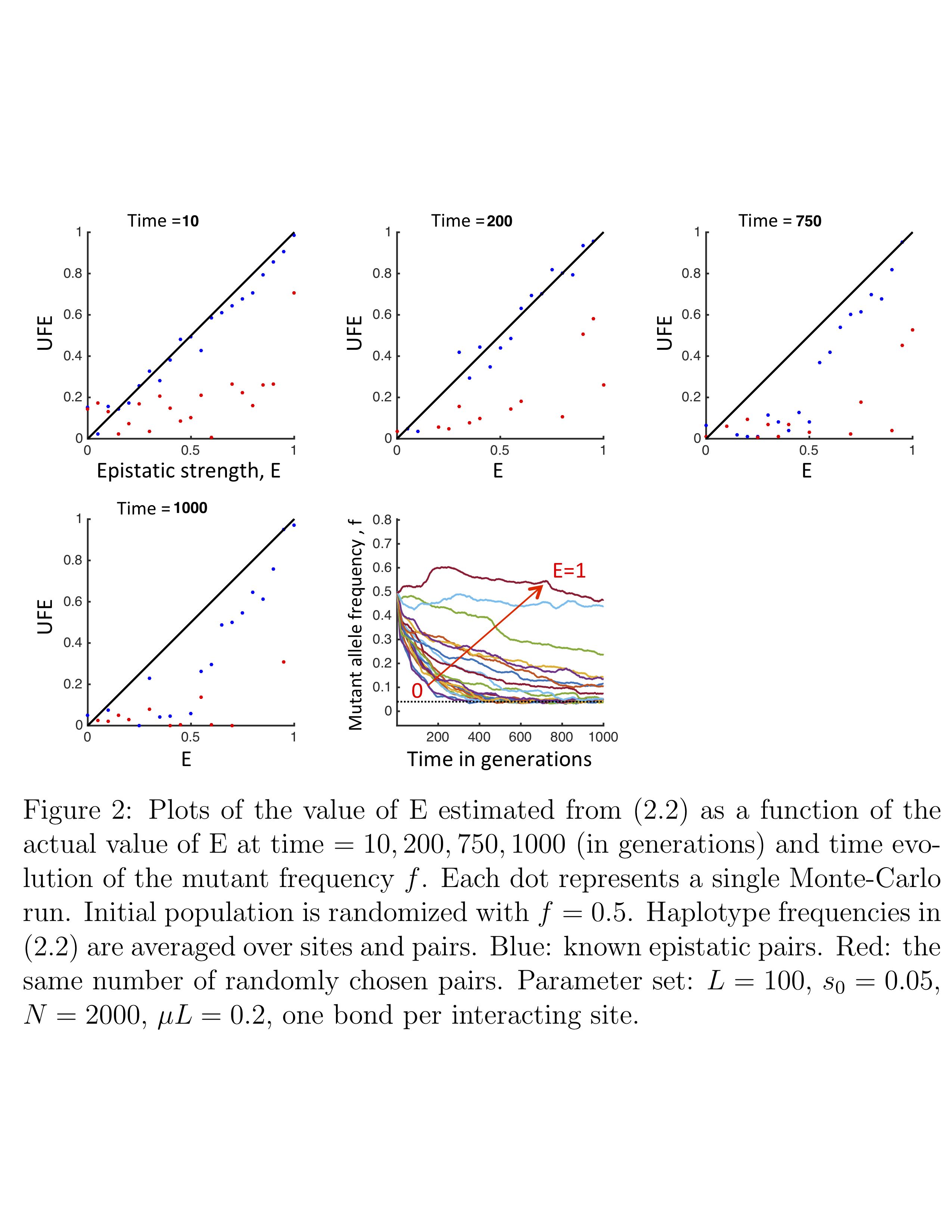

Supplement: S2 Fig — Shown are times in generations. Bottom right: Time evolution of the mutant frequency f. Each dot represents a single Monte-Carlo run. Initial population is randomized with f = 0.5. Haplotype frequencies in Eq (2.2) are averaged over sites and pairs. Blue: known epistatic pairs. Red: the same number of randomly chosen pairs. Parameter set: L = 100, s0 = 0.05, N = 2000, μL = 0.2, one bond per interacting site. (TIFF) [file pcbi.1006426.s005.tiff]

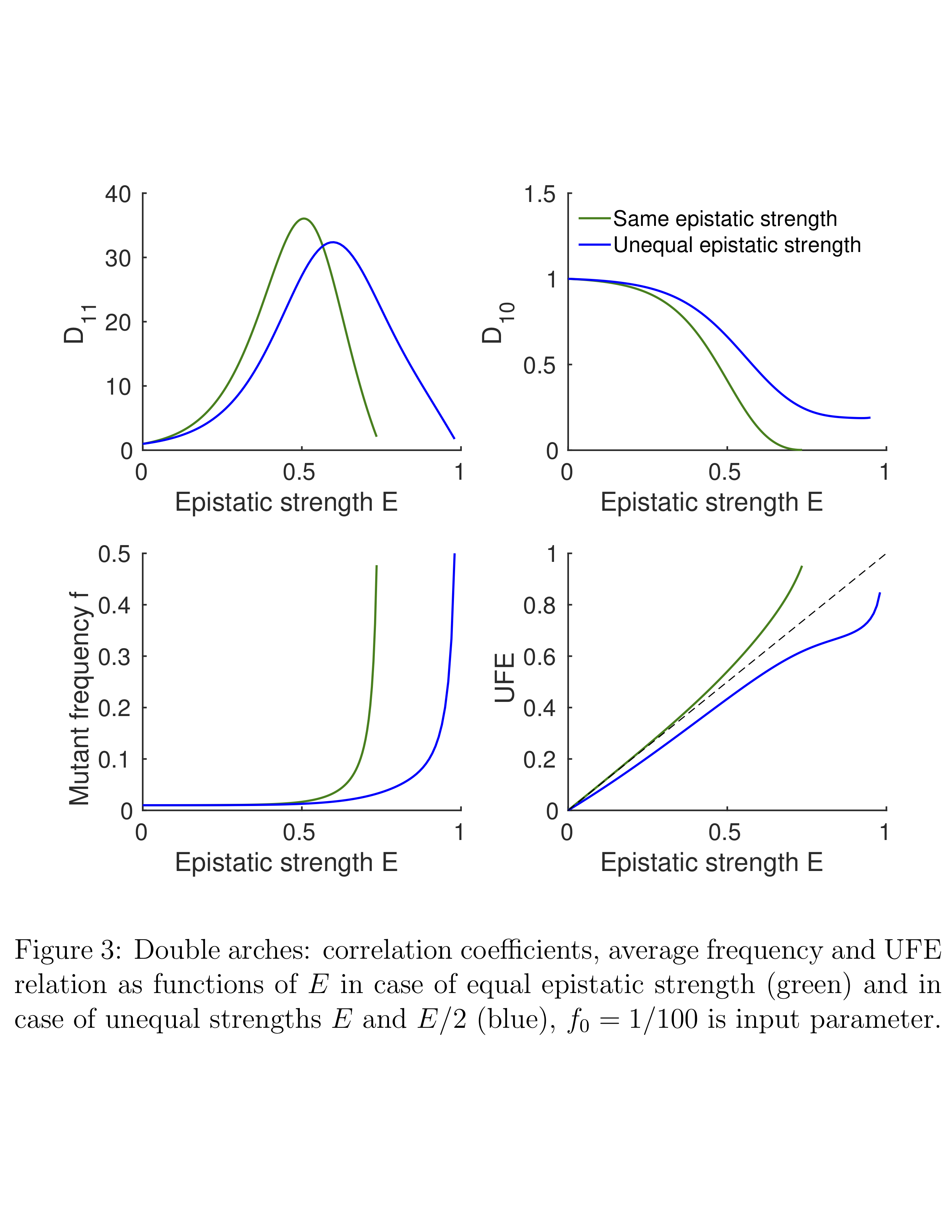

Supplement: S3 Fig — Shown are correlation coefficients, average frequency and UFE as a function of E in the case of equal epistatic strength (green) and in the case of unequal strengths E and E/2 (blue), f0 = 1/100 is input parameter. (TIFF) [file pcbi.1006426.s006.tiff]
